# Preclinical Development and Phase I Study of ZSYY001, a Polymeric Micellar Paclitaxel for Advanced Solid Tumor

**DOI:** 10.1002/cam4.71039

**Published:** 2025-07-22

**Authors:** Ge Gao, Pei Shu, Youzhen Tan, Tongsen Zheng, Wenmao Fan, Luding Lu, Huan Zhou, Zishu Wang, Ling Liu, Zhiqiang Liu, Yongsheng Wang

**Affiliations:** ^1^ Clinical Trial Center, National Medical Products Administration key Laboratory for Clinical Research and Evaluation of Innovative Drugs West China Hospital, Sichuan University Chengdu Sichuan China; ^2^ Division of Abdominal Tumor Multimodality Treatment Cancer Center, West China Hospital, Sichuan University Chengdu Sichuan China; ^3^ Guangdong Zhongsheng Pharmaceutical Co. Ltd. Guangzhou Guangdong China; ^4^ Department of Gastrointestinal Medical Oncology Harbin Medical University Cancer Hospital Harbin Heilongjiang China; ^5^ Department of Phase 1 Trials Center North Guangdong People's Hospital Guangzhou Guangdong China; ^6^ Department of Oncology North Guangdong People's Hospital Guangzhou China; ^7^ Department of Phase 1 Trials Center First Affiliated Hospital of Bengbu Medical College Bengbu Anhui China; ^8^ Department of Oncology First Affiliated Hospital of Bengbu Medical College Bengbu Anhui China; ^9^ Division of Thoracic Tumor Multimodality Treatment Cancer Center, West China Hospital, Sichuan University Chengdu Sichuan China

## Abstract

**Purpose:**

A novel paclitaxel delivery system has the potential to avoid the side effects associated with Cremophor EL and thus enhance therapeutic efficacy. Here, we report the results of a preclinical and phase I clinical study investigating ZSYY001, a nanoparticle formulation of polymeric micellar paclitaxel (PM‐paclitaxel).

**Methods:**

In preclinical studies, A549, MDA‐MB‐231, and SKOV3 xenograft tumor models were developed. Various concentrations of ZSYY001 were administered, and tumor growth and body weight were measured. Sprague–Dawley (SD) rats and Beagle dogs were used to evaluate the toxicity. In the phase I study, a dose‐escalation study using a 3 + 3 design was conducted in patients with solid tumors. The PM‐paclitaxel dose was escalated from 175 mg/m^2^ to 390 mg/m^2^. PM‐paclitaxel was intravenously administered over 3 h every 21 days without any premedication. This study was registered with number CTR20210347.

**Results:**

Preclinical studies showed that ZSYY001 significantly inhibited tumor growth without causing weight loss. In the phase I study, all patients were evaluable for toxicity and pharmacokinetic analysis, and 18 patients were evaluable for tumor response. Acute hypersensitivity reactions were not observed. Anemia and hair loss were the most common toxicities. Dose‐limiting toxicity events were not observed at any dose levels. Dose escalation to 390 mg/m^2^ did not identify a maximum‐tolerated dose. There were two partial responses (11.11%) and seven cases of stable disease (38.89%) among the 18 patients, five of whom had prior exposure to paclitaxel chemotherapy. The paclitaxel area under the curve and peak paclitaxel concentration suggest that PM‐paclitaxel does not exhibit linear pharmacokinetics.

**Conclusions:**

ZSYY001 was safe and well tolerated without additional toxicity and exhibited desirable antitumor activity, making it a promising treatment.

**Trial Registration:**

CTR20210347

## 1 Introduction

Paclitaxel is a well‐established chemotherapeutic drug used for the treatment of various solid tumors [1]. To address its poor water solubility, Cremophor EL (CrEL) was added [2]. However, CrEL can cause allergic reactions, necessitating the pre‐administration of dexamethasone and diphenhydramine, limiting the dosage and extending infusion time [1, 2]. To eliminate the need for CrEL, polymer micelle paclitaxel was developed. Polymer micelle paclitaxel is a novel drug delivery system wherein paclitaxel is encapsulated within nanoscale particles, enhancing its ability to penetrate tumors. This system leverages the enhanced permeability and retention effect for passive targeting of tumor tissues, allowing for a significantly increased dosage of paclitaxel and improving its efficacy and safety [3, 4].

ZSYY001 is a polymeric micellar formulation of paclitaxel, composed of a block copolymer of methoxypolyethylene glycol and lactide. To assess its safety and efficacy, ZSYY001 was evaluated preclinically in BALB/c nude mice, Sprague–Dawley rats, and Beagle dogs, as well as clinically in a phase I study involving patients with solid tumors. The primary objective of this clinical study was to assess the safety and effects of ZSYY001 on solid tumors. Here, we report the results from both preclinical and phase I clinical studies of ZSYY001, which demonstrated: (1) inhibition of tumor growth in preclinical studies using A549, MDA‐MB‐231, and SKOV3 xenograft models, (2) favorable tolerability in toxicological safety evaluations using SD rats and Beagle dogs, and (3) a disease control rate of 50%, with acceptable safety observed in the clinical study.

## 2 Materials and Methods

### 2.1 Materials

PM‐paclitaxel (ZSYY001) and PM (nanocarrier) were provided by Guangdong Zhongsheng Pharmaceutical Co. LTD. Paclitaxel liposomes were purchased from Nanjing Luye Pharmaceutical Co. LTD., and paclitaxel was purchased from Haikou Qili Pharmaceutical Co. LTD. Paclitaxel and PM‐paclitaxel were dissolved in 0.9% normal saline, and paclitaxel liposomes were dissolved in 5% glucose. All dissolved solutions were stored below 25°C.

### 2.2 In Vivo Anti‐Tumor Efficacy of ZSYY001

For in vivo efficacy experiments, 18–20 g (±20%) and 4–6 weeks old female BALB/c nude mice (Sino‐British SIPPR/BK Lab. Animal Ltd., Shanghai) were used. MDA‐MB‐231, A549, and SKOV3 cell lines were purchased from the National Collection of Authenticated Cell Cultures (NCACC) (Shanghai, China). MDA‐MB‐231 cells (1 × 10^6^ cells/0.1 mL), A549 cells (6 × 10^6^ cells/0.1 mL), and SKOV3 cells (6 × 10^6^ cells/0.1 mL) were injected into the right flank of the BALB/c nude mice. When the tumor volume reached 100–200 mm^3^, tumor‐bearing mice were randomly grouped (*n* = 8–10) [Control (Ctrl): 0.9% normal saline; Paclitaxel liposomes: 20 mg/kg; Paclitaxel: 20 mg/kg; ZSYY001: 20 mg/kg; ZSYY001: 40.0 mg/kg] at equivalent dosages according to body weight. Each mouse received treatment every 3 days on Days 0, 3, 6, 9, 12, 15, and 18. Tumor volume and body weight were measured twice a week. Tumor volume was calculated as length×width^2^/2. The percentage change in body weight of the tumor‐bearing animals was calculated as follows: (weight at measurement−weight at grouping)/weight at grouping × 100%. All animal experiments were performed according to Institutional Animal Care and Use guidelines.

### 2.3 In Vivo Toxicity Study

SD rats were purchased from Beijing Vital River Laboratory Animal Technology Co. Ltd. The weight range was 237.4–265.9 g for the male rats and 164.2–194.7 g for the female rats. For acute toxicity studies, rats were randomly divided into six groups (*n* = 6–10) [Ctrl: 0.9% normal saline; PM: 1000 mg/kg; Paclitaxel: 98 mg/kg; ZSYY001 (low): 50 mg/kg; ZSYY001 (medium): 100 mg/kg; ZSYY001 (high): 200 mg/kg] with an equal number of males and females. For the assessment of subacute toxicity, the rats were randomly divided into six groups (*n* = 10) [Ctrl: 0.9% normal saline; PM: 120 mg/kg; Paclitaxel:4 mg/kg; ZSYY001 (low): 10 mg/kg; ZSYY001 (medium): 20 mg/kg; ZSYY001(high): 30 mg/kg] with an equal number of males and females. Beagle dogs were purchased from Shanghai Xingang Experimental Animal Farm, with a weight range of 8.0–12.0 kg. For acute toxicity studies, dogs were assigned to six groups (*n* = 6) [Ctrl: 0.9% normal saline; PM: 120 mg/kg; Paclitaxel: 8.0 mg/kg; ZSYY001 (low): 6 mg/kg; ZSYY001 (medium): 12 mg/kg; ZSYY001(high): 24 mg/kg]. For the assessment of subacute toxicity, dogs were assigned to six groups (*n* = 6) [Ctrl: 0.9% normal saline; PM: 60 mg/kg; Paclitaxel:2 mg/kg; ZSYY001 (low): 3 mg/kg; ZSYY001 (medium): 6 mg/kg; ZSYY001(high): 12 mg/kg]. For acute toxicity studies, each rat/dog received the appropriate treatment once and was observed continuously for 2 weeks. For subacute toxicity studies, each rat/dog received the appropriate treatment as above for the first 4 weeks and was then allowed to recover for 4 weeks. Body weight, survival, clinical observations, hematological, and biochemical indices were collected and recorded (Figure S1).

### 2.4 Phase I Study Population

Phase I study was performed at six centers in China. Eligible patients had histopathological and/or cytologically confirmed advanced solid malignancies recurrent to or failing standard treatments with paclitaxel monotherapy indications. The inclusion criteria included the following: (a) aged 18–70 years; (b) an Eastern Cooperative Oncology Group (ECOG) performance status of 0–1; (c) expected survival period of at least 3 months; (d) according to the Response Evaluation Criteria in Solid Tumors (RECIST) version 1.1, the patient has measurable lesions; (e) adequate hematological, renal, and hepatic function; (f) for the first administration in this study, toxic reactions from prior anticancer treatments must have recovered to grade ≤ 1 according to Common Terminology Criteria for Adverse Events (CTCAE) version 5.0 (excluding hair loss).

The main exclusion criteria included the following: (a) uncontrolled brain metastasis; (b) prior chemotherapy, immunotherapy, or radiation therapy for a period of 4 weeks; (c) grade ≥ 2 peripheral neuropathy; (d) grade ≥ 2 active bacterial infections; (e) the presence of psychiatric disease; (f) the presence of bronchial asthma, uncontrolled cardiac disease, uncontrolled diabetes, uncontrolled pleural effusion, pericardial effusion, and peritoneal effusion; (g) presence of hepatitis B, hepatitis C, or HIV positive. All the patients gave written informed consent according to national and institutional guidelines before therapy.

### 2.5 Phase I Study Design

The dose escalation study was designed to evaluate the tolerability, safety, PK, and pharmacodynamics (PD) in patients with advanced solid tumors. Patients were assigned sequentially to escalating dose levels of ZSYY001 following a traditional 3 + 3 design. The study included 5 ascending cohorts receiving ZSYY001 at 175 mg/m^2^, 230 mg/m^2^, 300 mg/m^2^, 360 mg/m^2^ and 390 mg/m^2^, respectively. All the patients in the dose escalation cohorts participated in the PK/PD study.

If a subject in each dose group does not experience DLT or any other adverse events deemed significant by the investigator within the first treatment cycle (D1~D21), the subject will continue to receive treatment with the same dose of ZSYY001. Alternatively, even if a DLT occurs during the first treatment cycle, if the investigator deems that the benefit to the subject outweighs the risks, the treatment with ZSYY001 may be continued with a reduced dose, with a maximum observation period of not more than six cycles during the entire dose escalation phase.

### 2.6 Safety Assessment

Safety evaluation included assessment for adverse events (AEs), vital signs, physical examination, ECOG score, body weight, laboratory examination (routine blood test, blood biochemistry, routine urine test, coagulation examination), 12‐lead electrocardiogram, and blood pregnancy examination for women of childbearing age during and after treatment. Details of AEs, including the description of AEs and all related symptoms, occurrence time, severity, causes of AEs, correlation with the test drug, duration, treatments, final results, and outcomes, were recorded. The timeframe for AE collection extends from the time the subject receives ZSYY001 until the end of the last follow‐up period (21 ± 7 days after the last treatment, or death, or initiation of other anti‐tumor therapy).

During the dose‐escalation phase in the first cycle (D1~D21), if a dose group has ≥ 2 out of 6 evaluable subjects experiencing DLT, that dose level is defined as intolerable. The MTD is defined as the dose level below the dose at which DLT is observed in two or more subjects, or the highest dose at which DLT is observed in ≤ 1 subject. If dose escalation continues to the highest dose without observing any DLT, then the highest dose is determined as MTD. A total of six evaluable subjects are required to define the MTD.

DLT was defined as any adverse events occurring during the first cycle (D1~D21) that are definitely related, or probably related to ZSYY001, including grade three febrile neutropenia, Grade 4 neutropenia lasting at least 4 days, Grade 3 thrombocytopenia with significant clinical bleeding symptoms, or Grade 4 thrombocytopenia, Grade 4 anemia, and Grade ≥ 3 non‐hematological toxicities other than nausea, vomiting, or diarrhea that can be controlled with treatment.

### 2.7 Efficacy Assessment

Computer tomography or magnetic imaging was performed as screening for every two administration cycles during treatment and at the end of the study visit. Solid tumor assessment was based on Response Evaluation Criteria in Solid Tumors (RECIST) version 1.1. The efficacy assessment metrics include objective response rate (ORR), progression‐free survival (PFS), duration of response (DOR), disease control rate (DCR), and overall survival (OS).

### 2.8 Pharmacokinetic Assessment

Pharmacokinetic studies were performed in all patients during the first administration cycle of ZSYY001. Blood samples (4 mL each) for PK analysis were collected at 14 time points during Cycle 1, Day 1: pre‐infusion(0 h, baseline), 0.5 h into infusion, and post‐infusion at 0 h, 10 min, 0.5, 1, 2, 4, 6, 8, 12, 24, 48, and 72 h. The concentration of free paclitaxel and total paclitaxel in plasma samples was determined using a validated liquid chromatography–tandem mass spectrometry (LC/MS/MS) method [5, 6, 7, 8, 9]. D5‐labeled paclitaxel was added to the plasma samples as the internal standard. Calibration curve ranges were linear over the range of 1.00–800 ng/mL for free paclitaxel and 15.0–12,000 ng/mL for total paclitaxel. The lower limit of quantification (LLOQ) for free and total paclitaxel was 1.00 ng/mL and 15.0 ng/mL, respectively. Parameters analyzed included: maximum observed plasma concentration (C_max_), time of maximum observed plasma concentration (T_max_), area under the plasma concentration‐time curve (AUC) for various time intervals (0‐t, 0‐∞), terminal elimination rate constant (λz), terminal elimination half‐life (t_1/2_), total body clearance (CL), apparent volume of distribution (Vd), and mean residence time (MRT).

### 2.9 Statistical Analysis

Quantitative data were described using mean, standard deviation. Categorical and ordinal data were described using frequency and percentages. Regarding the statistical analysis of intergroup differences in hematological, coagulation, and biochemical parameters, the statistical methods involved included Kolmogorov–Smirnov Test, Levene's test, ANOVA with Dunnett's post hoc test, Dunnett's T3 test, and the Mann–Whitney *U*‐test. The statistical analysis was performed as follows:Groups with sample sizes < 3 were excluded from the analysis. For parameters with sample sizes ≥ 3 in each group, normality was assessed using the Kolmogorov–Smirnov Test. Data passing the normality test (*p* ≥ 0.05) were further evaluated for homogeneity of variances using Levene's Test. Normally distributed data with homogeneous variances (*p* ≥ 0.05): ANOVA followed by Dunnett's post hoc test (for comparisons between treatment groups and the 0.9% NS group). Normally distributed data with heterogeneous variances (*p* < 0.05):Dunnett's T3 test (adjusted for variance heterogeneity) was applied. Non‐normally distributed data (One‐Sample Kolmogorov–Smirnov Test *p* < 0.05):Mann–Whitney *U*‐test was used for pairwise comparisons between treatment and control groups. All tests were two‐tailed with a significance level of *α* = 0.05. For group comparison of tumor volumes in preclinical anti‐tumor efficacy, unpaired Student's *t*‐test was used. *p*‐value < 0.05 indicated a significant difference between groups.

In phase I clinical trial, the PK parameter calculation for this study was performed using Phoenix WinNonlin (Pharsight Corporation, 8.3), and other analyses were conducted using SAS (version 9.4) software. The linear relationship between single‐dose PK parameters (C_max_, AUC_0–t_, AUC_0–∞_) and administered doses was evaluated using the power model confidence interval(CI) method. The regression equation was expressed as In(PK) = β0 + β1 × ln (dose). The (1−*α*) × 100% CI for the slope β1 was calculated, where *α* represents the significance level. In this study, *α* was set at 0.1, meaning that a 90% CI for β1 was computed. The predetermined CI criterion was set as follows: [1 + In(0.8)/In(*r*); 1 + In(1.25)/In(*r*)], where r represents the ratio of highest dosage(390 mg/m^2^)/lowest dosage(175 mg/m^2^). The linear pharmacokinetic judgment interval in this study was [72.15, 127.85]. If the 90% CI for β1 falls entirely within the predetermined CI, it is considered that the PK of ZSYY001 exhibits dose proportionality. Progression‐free survival analysis was performed using a Kaplan–Meier survival curve and a log‐rank test comparing each group.

## 3 Results

### 3.1 Preclinical Anti‐Tumor Efficacy of ZSYY001

**FIGURE 1:**
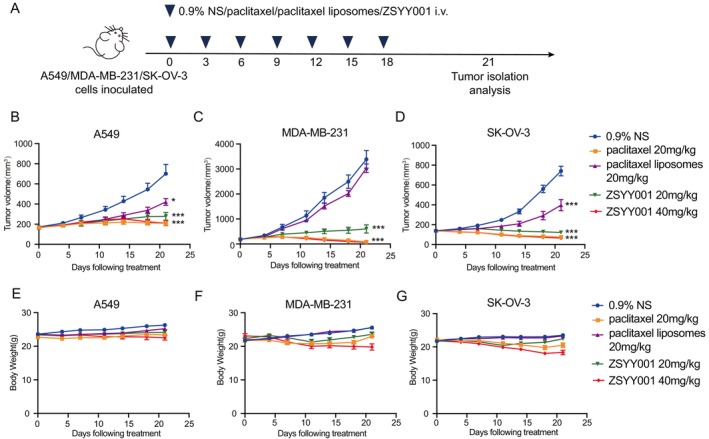
Broad anti‐tumor effect of ZSYY001 on diverse tumor models. (A) Schematic of the treatment schedule. Nude Balb/c mice were subcutaneously inoculated with A549 cells, MDA‐MB‐231 cells or SK‐OV‐3 cells (1 × 10^6^/mouse) and randomly grouped when the tumor volume come to 100~200 mm^3^ (± 10%). On Days 0, 3, 6, 9, 12, 15, and 18, 0.9% normal saline (0.9% NS), paclitaxel (20 mg/kg/mice), paclitaxel liposomes (20 mg/kg/mice), ZSYY001 (20 mg/kg/mice) and ZSYY001 (20 mg/kg/mice) were intravenous injected to mice in different groups (*n* = 8–10 mice pooled). (B–D) Tumor volumes were measured and recorded with a vernier caliper every 2 days. Tumor growth of A549 (B), MDA‐MB‐231 (C) and SKOV3 (D) were plotted starting from the day before initial dose of treatment (*n* = 7–10 mice pooled). Data were from one of two independent experiments. Each point represents the mean tumor volume and SEM. (E–G) Body weight (*n* = 7–10) of A549 (E), MDA‐MB‐231 (F) and SKOV3 (G) tumors in mice given different treatment. *t*‐test was used for comparison between groups. *p ≤ 0.05, compared with 0.9% normal saline group;**, *p* ≤ 0.01, compared with 0.9% normal saline group; ***, *p* ≤ 0.001, compared with 0.9% normal saline group.

To investigate whether PM‐paclitaxel ZSYY001 exhibits superior anti‐tumor effects compared to paclitaxel liposomes or paclitaxel, mice bearing human non‐small cell lung cancer (NSCLC) cells A549, human breast cancer cells MDA‐MB‐231, and human ovarian cancer cells SKOV3 xenograft tumor models were established (Figure 1A). ZSYY001 demonstrated superior tumor growth inhibition in xenograft tumor models derived from MDA‐MB‐231, A549, and SKOV3 cells, compared to paclitaxel and paclitaxel liposomes (Figure 1B–D). Mice tolerated doses of 20 mg/kg for paclitaxel, ZSYY001, and paclitaxel liposomes without significant changes in body weight across various tumor models. However, at a higher dose of 40 mg/kg, ZSYY001 had a noticeable impact on body weight (Figure 1E–G). These results highlight the significant tumor inhibition and broad applicability of ZSYY001 in solid tumors, though the toxicity profile at higher doses remains unclear.

### 3.2 Toxicological Safety Evaluation of ZSYY001 In Vivo

**FIGURE 2:**
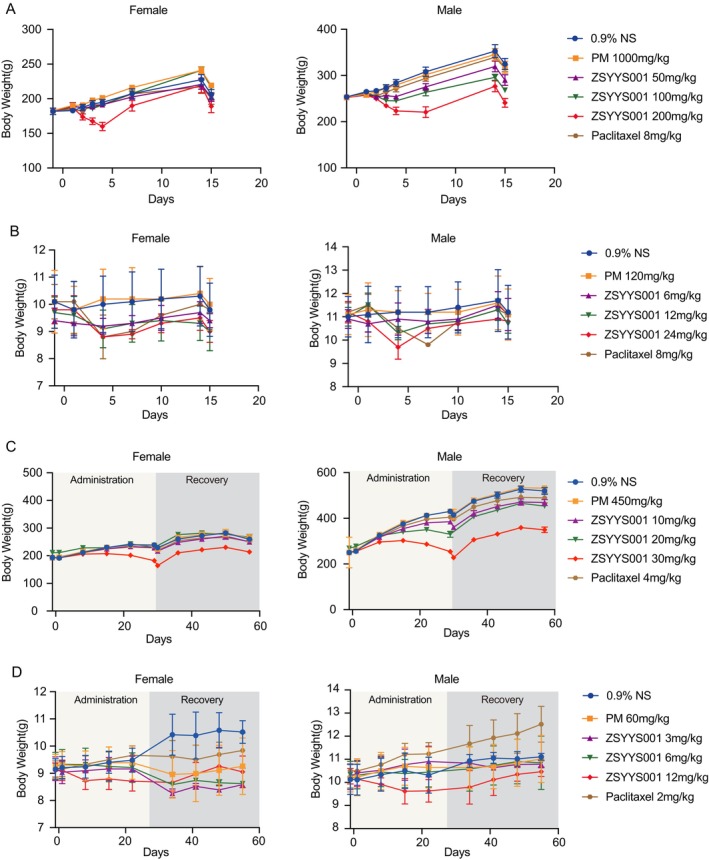
Acute and subacute toxicity of SD rats and Beagle dogs. (A) Body weight change of female(left) and male (right) SD rats in acute toxicity experiments. (B) Body weight change of female(left) and male (right) Beagle dogs in acute toxicity experiments. (C) Body weight change of female(left) and male (right) SD rats in subacute toxicity experiments. (D) Body weight change of female(left) and male (right) Beagle dogs in subacute toxicity experiments.

We further performed acute and subacute toxic effects of ZSYY001 on healthy SD rats and beagle dogs. SD rats treated with ZSYY001 showed a dose‐dependent decrease in body weight, particularly at 200 mg/kg (Figure 2A). Severe toxicity was observed in the 200 mg/kg ZSYY001 group from Day 4, including soft stools, watery diarrhea, reduced activity, abnormal gait, and a high mortality rate (5 out of 10 rats). In contrast, rats treated with paclitaxel (8 mg/kg) exhibited acute allergic reactions such as reduced activity and loss of righting reflex, which resolved by Day 2. Hematological and biochemical indices indicated mild anemia, decreased lymphocytes, and decreased alkaline phosphatase (ALP) in rats treated with ZSYY001 at doses of 50–200 mg/kg (Tables S1 and S2).

In the acute toxicological safety evaluation of ZSYY001 in Beagle dogs, it was found that the lethal dose of ZSYY001 is 24 mg/kg, at which significant weight loss was observed (Figure 2B), leading to the death of one animal. In comparison, the lethal dose of paclitaxel is 8 mg/kg, which resulted in the death of two animals. The main adverse reactions at high doses of ZSYY001 (24 mg/kg) and paclitaxel (8 mg/kg) included hair loss, vomiting, eye closure, soft stools, watery diarrhea, reduced activity, and loss of appetite. Additionally, animals in the paclitaxel group experienced allergic reactions within 17 min of administration. Hematological and biochemical indices indicated anemia, leukopenia, thrombocytopenia, decreased lymphocytes, and elevated levels of alanine aminotransferase (ALT), aspartate aminotransferase (AST), and creatine kinase (CK) in both the ZSYY001 (24 mg/kg) and paclitaxel (8 mg/kg) groups (Tables S3 and S4).

In subacute toxicity experiments conducted on both SD rats and Beagle dogs, animals were treated once a week for 4 weeks, followed by a 4‐week recovery period. In SD rats, no mortality was observed at any dose levels of ZSYY001 or paclitaxel. The main adverse reactions to ZSYY001 included hair loss, unsteady gait, muscle weakness, and weight loss (Figure 2C). Hematological and biochemical analyses showed significant reductions in neutrophils and lymphocytes, along with marked increases in ALT and AST levels (Tables S5 and S6). In contrast, no significant abnormalities in indicators or organ functions were observed in the paclitaxel group (4 mg/kg). In summary, the lowest observed adverse effect level (LOAEL) for ZSYY001 in SD rats was determined to be 10 mg/kg, and the maximum tolerated dose (MTD) was 30 mg/kg, which corresponds to 180 mg/m^2^ when converted based on body surface area.

In Beagle dogs, animals in the paclitaxel group exhibited acute allergic reactions, including skin redness, edema, and head shaking. Both ZSYY001 and paclitaxel caused symptoms such as hair loss, soft stools, watery stools, and vomiting. Animals in the high‐dose ZSYY001 group (12 mg/kg) experienced significant weight loss, which gradually recovered after the medication was discontinued (Figure 2D). Hematological and biochemical analyses showed reductions in neutrophils and white blood cells (WBC) in the ZSYY001 groups (Tables S7 and S8). In summary, the LOAEL for ZSYY001 in Beagle dogs was found to be 3 mg/kg, and the MTD was 12 mg/kg, corresponding to 230 mg/m^2^ when converted based on body surface area. The overall acute and subacute toxicological safety evaluation indicates that the toxicity induced by high doses of ZSYY001 is intolerable, while the toxicity at low and medium doses is potentially acceptable. Additionally, in both animals, the tolerated dose of ZSYY001 was significantly higher than that of paclitaxel.

### 3.3 Patient Characteristics of Phase I Study

**FIGURE 3:**
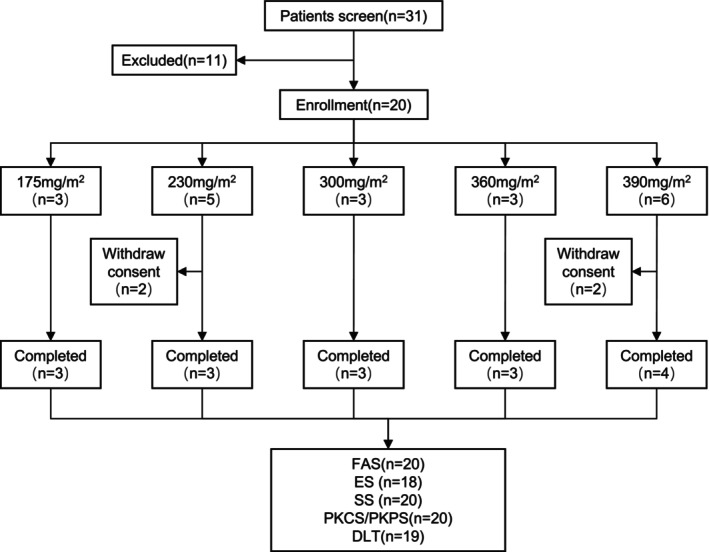
Flow diagram of screening eligible patients for inclusion. FAS, full analysis set; according to the intention‐to‐treat principle, all participants enrolled who received the study drug at least once were included. ES, efficacy set; subject population that can be evaluated for efficacy should meet the requirements of participants who have undergone imaging evaluation at baseline, received at least one investigational drug, and had at least one post‐administration tumor evaluation. SS, safety set; all subjects who received the study drug and had a record of post‐administration safety indicators were included. PKCS, pharmacokinetic concentration analysis set; all participants enrolled who received the study drug at least once and had at least one effective blood concentration data. PKPS, pharmacokinetic(PK) parameter analysis set; all participants enrolled who received the study drug at least once and had at least one PK parameter.

Between May 2021 and December 2022, 31 subjects were screened for the phase I study, with 20 subjects eventually enrolling across different dose groups: 175, 230, 300, 360, and 390 mg/m^2^, with 3, 5, 3, 3, and 6 subjects in each group, respectively. All of these subjects received the study drug at least once, with 16 subjects completing the trial and 4 withdrawing their informed consent and dropping out (Figure 3). The study drug, ZSYY001, was administered without antiemetic treatment or premedication with dexamethasone and histamine blockers. The characteristics of the patients are listed in Table 1. The median age was 51.5 years (range: 32–65), with 10 male and 10 female patients. Eight patients had lung cancer, four had breast cancer, two had gastric cancer, one had esophageal cancer, and the remaining five had other types, including thymic cancer and ovarian cancer. All of the patients had previously undergone chemotherapy, with most receiving three or more chemotherapy regimens, and 13 (65%) of them had received prior taxane therapy.

**TABLE 1:** Patients baseline characteristics.

No. of patients	20
Median age (range)	51.5 (32–65)
Sex, *n* (%)	
Male	10 (50)
Female	10 (50)
ECOG performance status, *n* (%)	
0	7 (35)
1	13 (65)
Tumor type, *n* (%)	
Non‐small cell lung cancer	8 (40)
Breast cancer	4 (20)
Esophageal carcinoma	1 (5)
Gastric carcinoma	2 (10)
Others	5 (25)
Prior chemotherapy, *n* (%)	
Yes	20 (100)
Prior taxane therapy	
Yes	13 (65)
No	7 (35)
Allergic history	
Yes	1 (5)
No	19 (95)

Abbreviation: ECOG, Eastern Cooperative Oncology Group.

### 3.4 Safety and Tolerability

In the safety evaluation, all 20 patients experienced at least one treatment‐related adverse event (TRAE) (Table 2). Among them, 1 patient (5%) had Grade 1 events, 10 patients (50%) had Grade 2 events, 6 patients (30%) had Grade 3 events, and 3 patients (15%) had Grade 4 events. No Grade 5 adverse events were observed. Except for one patient in the 260 mg/m^2^ dose group who did not reach the DLT assessment time, no DLTs or MTD were observed in the other dose groups. Common TRAEs included anemia (70%), hair loss (70%), neutropenia (50%), leukopenia (50%), lymphopenia (50%), and musculoskeletal pain (45%). Grade 3–4 adverse events occurred in 9 patients (45%), with neutropenia (25%), lymphopenia (25%), and leukopenia (15%) being particularly common. Serious adverse events (SAEs) related to the drug occurred in 2 patients (10%), including 1 case of Grade 4 neutropenia (20%) and 1 case of Grade 2–3 elevated ALT and AST (20%). One patient in the 175 mg/m^2^ dose group experienced an SAE of cancer pain, which was considered unrelated to the drug. Dosing of ZSYY001 was interrupted in 1 patient (5%) in the 360 mg/m^2^ group due to a Grade 2 allergic reaction and reduced in 1 patient (5%) in the same group due to Grade 1 weight loss. No patients withdrew from the trial due to adverse events. Overall, ZSYY001 was considered to have tolerable safety, with adverse reactions primarily being hematological toxicity but all being manageable.

**TABLE 2:** Treatment related adverse events by dose level (SS).

AE	175 mg/m^2^ (*n* = 3)		230 mg/m^2^ (*n* = 5)		300 mg/m^2^ (*n* = 3)		360 mg/m^2^ (*n* = 3)		390 mg/m^2^ (*n* = 6)		Total *N* (%)
	G1/2	G3/4	G1/2	G3/4	G1/2	G3/4	G1/2	G3/4	G1/2	G3/4	
Anemia	2	0	3	0	1	0	3	0	5	0	14 (70)
Hair loss	3	0	4	0	2	0	3	0	2	0	14 (70)
Leukopenia	1	0	1	1	1	0	1	1	3	1	10 (50)
Neutropenia	1	0	0	2	2	0	1	1	1	2	10 (50)
Lymphopenia	0	0	0	2	1	0	2	0	4	1	10 (50)
Musculoskeletal pain^a^	0	0	1	0	3	0	2	0	2	0	9 (45)
Rash	1	0	0	0	2	0	1	0	3	0	7 (35)
Hypaesthesia	1	0	2	0	1	0	1	0	2	0	7 (35)
Fatigue	1	0	1	0	2	0	0	0	2	0	6 (30)
Bone marrow suppression	1	0	2	0	1	1	0	0	0	0	5 (25)
Paresthesia	0	0	0	0	2	0	2	0	1	0	5 (25)
Diarrhea	0	0	3	1	0	0	1	0	0	0	5 (25)
γ‐GGT increased	0	0	1	0	2	0	0	0	1	1	5 (25)
ALP increased	0	0	1	0	1	0	1	0	1	0	4 (20)
Nausea	0	0	2	0	1	0	0	0	1	0	4 (20)
Constipation	0	0	1	0	2	0	1	0	1	0	4 (20)
ALT increased	0	0	0	1	0	0	1	0	2	0	4 (20)
AST increased	0	0	0	1	0	0	0	0	2	0	3 (15)
Fever	0	0	2	0	1	0	0	0	0	0	3 (15)
Vomiting	1	0	0	0	1	0	1	0	0	0	3 (15)
Weight loss	1	0	0	0	1	0	1	0	0	0	3 (15)
Allergic reaction	0	0	1	0	0	0	1	0	1	0	3 (15)
Pruritus	1	0	1	0	0	0	0	0	0	0	2 (10)
Oral mucositis	0	0	1	0	0	0	0	0	1	0	2 (10)
Hypertriglyceridemia	0	0	0	0	1	0	1	0	0	0	2 (10)
Hypokalemia	0	0	2	0	0	0	0	0	0	0	2 (10)
Anorexia	0	0	0	0	2	0	0	0	0	0	2 (10)
Epistaxis	0	0	0	0	1	0	1	0	0	0	2 (10)
Cough	0	0	0	0	0	0	1	0	1	0	2 (10)
Sinus tachycardia	0	0	0	0	1	0	1	0	0	0	2 (10)

Abbreviations: γ‐GGT, gamma‐glutamyl transferase; AE, adverse event; ALP, alkaline phosphatase; ALT, alanine aminotransferase; AST, aspartate aminotransferase; SS, safety set.

^a^ Musculoskeletal pain includes joint pain, myalgia, and limb pain.

### 3.5 Anti‐Tumor Effect

**FIGURE 4:**
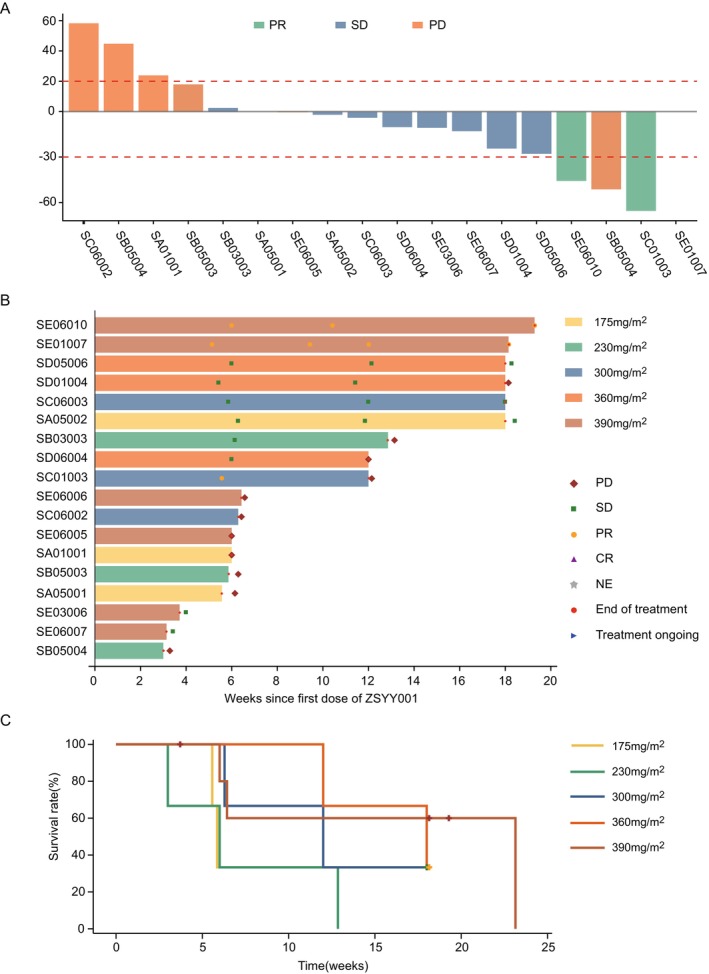
Tumor assessment of patients. (A) Waterfall plot of the maximum tumor change from baseline (most remarkable% tumor shrinkage). (B) Treatment duration and duration of response for individual patients. (C) Kaplan–Meier survival curves for progression‐free survival. CR, complete response; NE, not evaluable; PD, progressive disease; PM‐paclitaxel, polymeric micellar paclitaxel; PR, partial response; SD, stable disease.

Among the 18 patients evaluable for anti‐tumor response, 2 patients (11.11%) achieved a partial response (PR), both at the 390 mg/m^2^ dose level. The first patient experienced a 45.77% reduction in tumor size, with the partial response lasting for almost 5 months until the end of treatment. The second patient showed a 65.55% reduction after the first treatment course, maintaining this response until the end of the study. One patient treated at the 300 mg/m^2^ dose level initially showed a 51.3% reduction in tumor size during the first course, but the disease progressed by the second evaluation. Seven patients (38.89%) had stable disease, all of which were confirmed, while another seven patients (38.89%) experienced progressive disease as their best response. The most significant tumor size reductions and corresponding efficacy assessments are presented in Figure 4A,B. The ORR was 11.11%, and the DCR was 50% (Table 3). Among these nine patients, five had previously received paclitaxel chemotherapy. By the end of the study, no disease progression was observed in six patients, and the median PFS for the 12 evaluable subjects was 84 days (95% CI, 42 to 162) (Figure 4C).

**TABLE 3:** Summary of best overall response.

	175 mg/m^2^ (*n* = 3)	230 mg/m^2^ (*n* = 3)	300 mg/m^2^ (*n* = 3)	360 mg/m^2^ (*n* = 3)	390 mg/m^2^ (*n* = 6)	All (*N* = 18)
BOR, *n* (%)						
CR	0	0	0	0	0	0
PR	0	0	0	0	2 (33.33)	2 (11.11)
SD	1 (33.33)	1 (33.33)	2 (66.67)	3 (100.00)	0	7 (38.89)
PD	2 (66.67)	2 (66.67)	1 (33.33)	0	2 (33.33)	7 (38.89)
NE	0	0	0	0	2 (33.33)	2 (11.11)
ORR, *n* (%), (95% CI)	0	0	0	0	2 (33.33)	2 (11.11)
	(0.00,70.76)	(0.00,70.76)	(0.00,70.76)	(0.00,70.76)	(4.33,77.72)	(1.38,34.71)
DCR, *n* (%), (95% CI)	1 (33.33)	1 (33.33)	2 (66.67)	3 (100.00)	2 (33.33)	9 (50.00)
	(0.84,90.57)	(0.84,90.57)	(9.43,99.16)	(29.24,100.00)	(4.33,77.72)	(26.02,73.98)

Abbreviations: BOR, best overall response; CI, confidence interval; CR, complete response; DCR, disease control rate; NE, not evaluable; ORR, objective response rate; PD, progressive disease; PR, partial response; SD, stable disease.

### 3.6 PK Analysis

**FIGURE 5:**
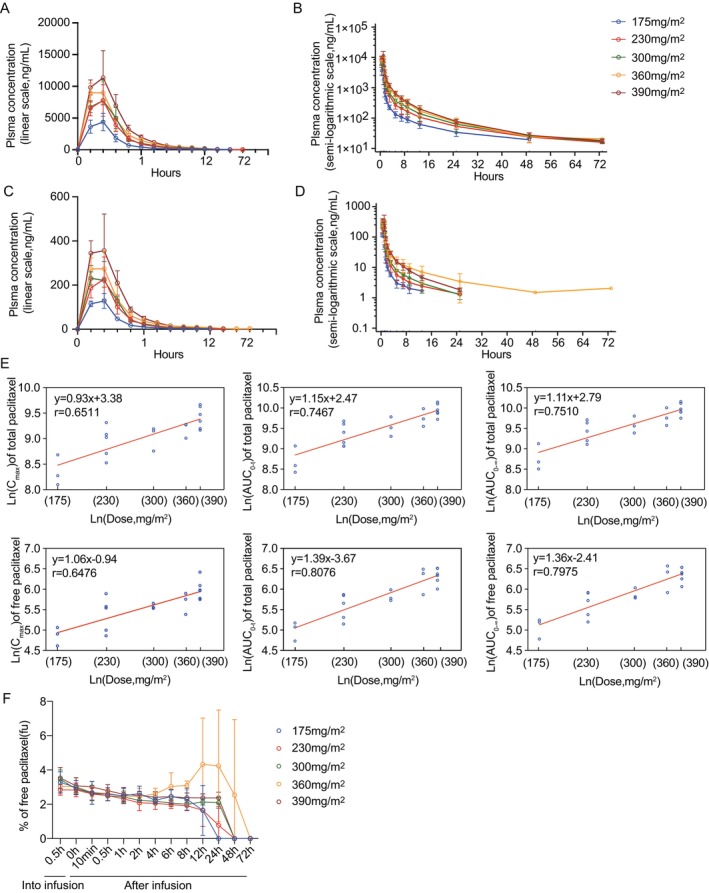
Plasma concentration–time profile of paclitaxel in each dose level. (A, B) Plasma concentration–time profile of total paclitaxel (A, linear scale; B, semi‐logarithmic scale). (C, D) Plasma concentration–time profile of free paclitaxel(C, linear scale; D, semi‐logarithmic scale). (E) Scatter plot of C_max_ versus dose, AUC_0−t_ versus dose and AUC_0–∞_ vs. dose of total and free paclitaxel. Data are mean ± SD (*n* = 3–6 per group). The ‘‐’ represent regression line. (F) Percentage of free paclitaxel(fu) in plasma at various times from start of the infusion.

All 20 patients were included in PK studies across a dose range of 175–390 mg/m^2^. As shown in Figure 5A–D, the concentration versus time curves for both total and free paclitaxel revealed that maximum plasma concentrations were achieved at the end of infusion, followed by a rapid decrease from C_max_. The PK parameters for total and free paclitaxel after administration are detailed in Table S9. It was observed that the mean C_max_, AUC_0‐t_, and AUC_0‐∞_ increased proportionally with the dosage within the 175–390 mg/m^2^ range. However, regression analysis based on the actual administered doses and corresponding PK parameters (C_max_, AUC_0‐t_, and AUC_0‐∞_) appeared to be nonlinear across the specific dose range(Figure 5E; Table S10). T_1/2_ of total paclitaxel after ZSYY001 administration fluctuated among different dose groups but generally remained within the range of 13.19–16.45 h. T_max_ varied little among dose groups, ranging approximately from 0.67 to 0.98 h. The median CL of ZSYY001 for dose levels ranged from 27.33 to 42.58 L/h. The Vd fluctuated significantly among dose groups but was consistently greater than 600 L, indicative of ZSYY001's extensive tissue binding. When plasma levels were high(0–8 h) the unbound fraction of paclitaxel in plasma, fu, was approximately 2%–4% and it almost remained unchanged within the dose range, except for the 360 mg/m^2^ dose group. In this group, a slightly elevated %fu was observed at 8 h and beyond, likely attributable to an isolated free drug measurement (Figure 5F). To validate this, we reanalyzed the data after excluding the outlier (Figure S2), which confirmed that %fu remained stable within the studied dose range.

## 4 Discussion

This, to our knowledge, was the first clinical study in which ZSYY001, a polymeric micellar paclitaxel, was used in patients with advanced solid tumors. Combining the data from preclinical and phase I clinical studies, it is indicated that ZSYY001 is well tolerated and effective.

As paclitaxel is a long‐established, broad‐spectrum chemotherapy drug, paclitaxel micelles were developed to solve the allergic reactions. Existing preclinical and clinical research has shown the effectiveness of paclitaxel micelles in patients with advanced solid tumor [4, 10, 11, 12, 13]. We propose that it is imperative to test whether ZSYY001, a paclitaxel micelle, could be safe and effective. This study aimed to assess the safety and efficacy of ZSYY001 through preclinical and phase I clinical studies.

In a preclinical study, we employed three different models and observed that ZSYY001 could inhibit tumor growth without causing weight loss. In three models, the inhibitory effect of polymer micelle paclitaxel at the same dosage was less than that of paclitaxel. However, increasing the dosage enhanced the inhibitory effect. At the same dosage, mice treated with polymer micelle paclitaxel experienced less weight loss compared to those treated with paclitaxel. Even with a higher dosage, no significant weight loss was observed, indicating better tolerability and lower toxicity for polymer micelle paclitaxel. Only in the human breast cancer MDA‐MB‐231 nude mouse xenograft model was the weight loss at the same dosage of polymer micelle paclitaxel for injection similar to that of paclitaxel injection; increased dosage resulted in more noticeable weight loss, but it remained tolerable. Moreover, at the same dosage, the tumor inhibitory effect of polymer micelle paclitaxel for injection was superior to that of paclitaxel liposomes. Observations on drug toxicity showed that the tolerable dose of paclitaxel was significantly lower than that of polymer micelle paclitaxel for injection, with the former causing more pronounced toxic reactions. Pharmacokinetic results indicated that the paclitaxel exposure in animals treated with paclitaxel injection (8 mg/kg) was significantly higher (approximately three times) than in those treated with polymer micelle paclitaxel (24 mg/kg), which could be one reason for the greater toxicity of paclitaxel. Additionally, severe acute allergic reactions, even leading to death, were observed in the paclitaxel after administration. These findings lay the foundation for conducting clinical trials.

The safety and toxicity of ZSYY001 were manageable for patients in the present study. No DLTs or unanticipated safety events were observed across the dose levels explored. Most reported TEAEs were predictable and predominantly Grade 1–2. The majority of Grade 3 or 4 TEAEs or TESAEs can be effectively managed through drug dosage adjustment and symptomatic support treatment. Dosing of ZSYY001 was interrupted in 1 (5%) patient in the 360 mg/m^2^ group. The safety and tolerability profile of ZSYY001 appears to be consistent with that of previously reported paclitaxel micelles [4, 14]. Previously reported paclitaxel micelles showed DLTs and MTD at 390 mg/m^2^ and 435 mg/m^2^ [14, 15], whereas no DLTs and MTD were observed in our study, further confirming that ZSYY001 is safe and tolerable.

Promising antitumor efficacy signals were observed in this study, especially in the 390 mg/m^2^ group. At present, there are two approved PM‐paclitaxel with ORRs of 14%–33.3% [4, 15]. In this study, the ORR in the 390 mg/m^2^ group was 33.3%. Several patients in the 390 mg/m^2^ group who had previously received alkylating agent chemotherapy also showed efficacy. The median PFS for the 390 mg/m^2^ dose group was 126.00 days. In the Phase I study of Genexol‐PM, PRs were observed in 3 of 21 patients (14%), distributed across the 175, 230, and 390 mg/m^2^ dose groups, with an overall DCR of 42%. The DCR of ZSYY001 (50%) was slightly higher than that of Genexol‐PM (42%), while the ORR values were comparable (11.11% vs. 14%). As a similar drug to Genexol‐PM, ZSYY001 has demonstrated comparable antitumor activity in the Phase I clinical study, warranting further exploration in Phase II clinical trials to confirm its efficacy and optimize dosing strategies.

Although pharmacokinetic parameters(C_max_, AUC_0‐t_, and AUC_0‐∞_) for both total and free paclitaxel showed an increasing trend with dose across the range of 175–390 mg/m^2^, the point estimates did not consistently fall within the confidence intervals. Concurrent dose‐dependent increases in apparent Vd and CL further indicated that the pharmacokinetics did not exhibit a linear relationship over this dosing range. Earlier studies of Genexol‐PM displayed linear dose‐proportionality as the dose escalated from 80 to 200 mg/m^2^ [16], but at higher doses, the drug also did not exhibit a linear pharmacokinetic profile [4]. One possible reason is that the drug‐loading capacity and binding affinity of micelles approach saturation as the dose escalates, leading to altered drug release kinetics and tissue distribution patterns [17]. In our study, during periods of high plasma concentrations (0–8 h post‐dose), the percentage of free paclitaxel (%fu) remained nearly stable (2%–4%) across the investigated dose range. This finding indicates that micellar drug release was relatively stable within the studied dose range, and plasma protein binding and drug‐loading capacity of polymeric micelles may remain unsaturable. Additionally, the observed dose‐dependent increases in Vd and CL were not driven by changes in %fu but may instead reflect intrinsic distribution and elimination mechanisms of the micellar carrier itself (e.g., enhanced tissue penetration or micelle‐specific clearance pathways). In Taxol's pharmacokinetic studies, high‐dose administrations exhibited nonlinear reductions in %fu, limiting dose escalation due to unmanageable toxicity (e.g., neurotoxicity, myelosuppression) [18]. Although the current micellar formulation does not achieve fully linear PK, the dose‐dependent changes in Vd and CL are primarily driven by carrier behavior rather than %fu dynamics, suggesting potential for optimization through carrier engineering. Additionally, factors such as micelle stability, saturation of drug‐metabolizing enzymes or receptors may also result in non‐linear changes in drug concentrations in vivo [17]. Although a linear pharmacokinetic relationship was not observed, the AUC_0‐∞_ and C_max_ of ZSYY001 generally showed an increasing trend with dose, with values in the same dose group slightly higher than those of equivalent doses of Genexol‐PM (notably lower in the 300 mg/m^2^ dose group) and significantly lower than those of Taxol [19]. The T_1/2_ (13.19–22.41 h) of ZSYY001 is comparable to that of Genexol‐PM (11.0–17.9 h), with both showing similar half‐lives. However, the average half‐life of ZSYY001 is shorter than that of Taxol at the 175 mg/m^2^ dose [19]. Overall, based on pharmacokinetic parameters, ZSYY001 exhibits more stable changes in C_max_ and AUC with dose adjustments compared to Genexol‐PM, which facilitates more precise dosing. The drug absorption and elimination of ZSYY001 are generally consistent with Genexol‐PM.

This study has several limitations. First, the number of enrolled patients was relatively small, limiting the power of the study. Second, the current study has not yet identified the optimal tumor type for treatment. With the promising results gained from this phase I clinical trial, a larger sample size and extended treatment duration for patients with advanced solid tumors are needed. Yet established formulations like Taxol (Cremophor EL‐based paclitaxel) and Abraxane (albumin‐bound paclitaxel) are widely used and serve as standard treatments in various cancers. Further pharmacokinetic comparison studies between ZSYY001 and paclitaxel and other phase II/III control comparisons with different paclitaxel formulations could potentially provide more clinically relevant insights, especially regarding hypersensitivity reactions, safety profiles, and overall efficacy.

In summary, we present the first reported clinical data on the use of ZSYY001, a polymeric micellar paclitaxel, in patients with advanced solid tumors. ZSYY001 had favorable safety and tolerability profiles and demonstrated good efficacy, consistent with preclinical studies.

## Author Contributions

Ge Gao: writing – original draft, review and editing, project administration. Pei Shu: writing – original draft, review and editing, project administration. Youzhen Tan: conceptualization, investigation. Tongsen Zheng: project administration. Wenmao Fan: project administration. Luding Lu: project administration. Huan Zhou: project administration. Zishu Wang: project administration. Ling Liu: data curation, formal analysis. Zhiqiang Liu: data curation, formal analysis. Yongsheng Wang: writing – review and editing.

## Ethics Statement

The study was approved by the institutional research ethics committee of the West China Hospital of Sichuan University, Chengdu, China.

## Consent

Written informed consent was obtained from all individual patients included in the study.

## Conflicts of Interest

This research is sponsored by Guangdong Zhongsheng Pharmaceutical Co. Ltd., in which Zhenyou Tan, Ling Liu, Zhiqiang Liu were the full time employee. These authors contributed to preclinical studies. Other authors declare that they have no known financial or non‐financial interests that are directly or indirectly related to the work submitted for publication.

## Supporting information


**Table S1.** ZSYY001‐induced hemopoietic toxicity in short‐term toxicity test of healthy SD rats (Day 15).
**Table S2.** ZSYY001‐induced changes of blood biochemical and coagulogram indexes in short‐term toxicity test of healthy SD rats (Day 15).
**Table S3.** ZSYY001‐induced hemopoietic toxicity in short‐term toxicity test of healthy Beagle dogs (Day 15).
**Table S4.** ZSYY001‐induced changes of blood biochemical and coagulogram indexes in short‐term toxicity test of healthy Beagle dogs (Day 15).
**Table S5.** ZSYY001‐induced hemopoietic toxicity in long‐term toxicity test of healthy SD rats (4 weeks).
**Table S6.** ZSYY001‐induced changes of blood biochemical and coagulogram indexes in long‐term toxicity test of healthy SD rats (4 weeks).
**Table S7.** ZSYY001‐induced hemopoietic toxicity in long‐term toxicity test of healthy Beagle dogs (4 weeks).
**Table S8.** ZSYY001‐induced changes of blood biochemical and coagulogram indexes in long‐term toxicity test of healthy Beagle dogs (4 weeks).
**Table S9.** Pharmacokinetic parameters of free and total paclitaxel in plasma after the first dose (Mean ± SD).
**Table S10.** Linear pharmacokinetic analysis (C_max_, AUC_0‐t_, AUC_0‐∞_) for total paclitaxel and free paclitaxel.

**Figure S1.** Toxicity profile of ZSYY001 in healthy SD rats and beagle dogs. (A) Sixty rats were allocated to six groups [Ctrl: 0.9% normal saline; PM: 1000 mg/kg; Paclitaxel: 8 mg/kg; ZSYY001 (low): 50 mg/kg; ZSYY001 (medium): 100 mg/kg; ZSYY001 (high): 200 mg/kg]. Thirty‐six beagles were allocated to six groups [Ctrl: 0.9% normal saline; PM: 120 mg/kg; Paclitaxel: 8.0 mg/kg; ZSYY001 (low): 6 mg/kg; ZSYY001 (medium): 12 mg/kg; ZSYY001(high): 24 mg/kg]. All animals received the corresponding administration once followed by 4 weeks observation. Macroscopic characteristics after ZSYY001‐induced acute toxicity were observed. (B) Sixty SD rats were allocated to six groups [Ctrl: 0.9% normal saline; PM: 120 mg/kg; Paclitaxel:4 mg/kg; ZSYY001 (low): 10 mg/kg; ZSYY001 (medium): 20 mg/kg; ZSYY001(high): 30 mg/kg]. Thirty‐six beagles were allocated to six groups [Ctrl: 0.9% normal saline; PM: 60 mg/kg; Paclitaxel:2 mg/kg; ZSYY001 (low): 3 mg/kg; ZSYY001 (medium): 6 mg/kg; ZSYY001(high): 12 mg/kg]. All animals received the corresponding administration once a week for 4 consecutive weeks and then did not receive administration for 4 weeks. Macroscopic characteristics after ZSYY001‐induced sub‐acute toxicity were observed.
**Figure S2.** Percentage of free paclitaxel(fu) in plasma at various times from start of the infusion. * Remove one isolated free drug measurement from the 360 mg/m^2^ dose group.

## Data Availability

The data that support the findings of this study are available from the corresponding author upon reasonable request.
