# Supplementary material for: Preclinical Development and Phase I Study of ZSYY001, a Polymeric Micellar Paclitaxel for Advanced Solid Tumor
Source: Cancer Med. 2025 Jul 22;14(14):e71039. doi: 10.1002/cam4.71039 (PMC12281596; doi:10.1002/cam4.71039)
Supplement: Supplementary file 1 — Table S1. ZSYY001‐induced hemopoietic toxicity in short‐term toxicity test of healthy SD rats (Day 15). Table S2. ZSYY001‐induced changes of blood biochemical and coagulogram indexes in short‐term toxicity test of healthy SD rats (Day 15). Table S3. ZSYY001‐induced hemopoietic toxicity in short‐term toxicity test of healthy Beagle dogs (Day 15). Table S4. ZSYY001‐induced changes of blood biochemical and coagulogram indexes in short‐term toxicity test of healthy Beagle dogs (Day 15). Table S5. ZSYY001‐induced hemopoietic toxicity in long‐term toxicity test of healthy SD rats (4 weeks). Table S6. ZSYY001‐induced changes of blood biochemical and coagulogram indexes in long‐term toxicity test of healthy SD rats (4 weeks). Table S7. ZSYY001‐induced hemopoietic toxicity in long‐term toxicity test of healthy Beagle dogs (4 weeks). Table S8. ZSYY001‐induced changes of blood biochemical and coagulogram indexes in long‐term toxicity test of healthy Beagle dogs (4 weeks). Table S9. Pharmacokinetic parameters of free and total paclitaxel in plasma after the first dose (Mean ± SD). Table S10. Linear pharmacokinetic analysis (Cmax, AUC0‐t, AUC0‐∞) for total paclitaxel and free paclitaxel. [file CAM4-14-e71039-s002.docx]

**Supplementary Table 1.** **ZSYY001-induced hemopoietic toxicity in short-term toxicity test of healthy SD rats(Day 15).**

| **Index** | **Units** | | **0.9% normal saline** | | **PM^a^**  **1000mg/kg** | | **ZSYY001**  **50mg/kg** | | **ZSYY001**  **100mg/kg** | | **ZSYY001**  **200mg/kg** | | **Paclitaxel**  **8mg/kg** | |
| --- | --- | --- | --- | --- | --- | --- | --- | --- | --- | --- | --- | --- | --- | --- |
|  |  |  | **Male**  **(n=5)** | **Female**  **(n=5)** | **Male**  **(n=5)** | **Female**  **(n=5)** | **Male**  **(n=5)** | **Female**  **(n=5)** | **Male**  **(n=5)** | **Female**  **(n=5)** | **Male**  **(n=2)** | **Female**  **(n=5)** | **Male**  **(n=5)** | **Female**  **(n=5)** |
| **RBC** | 10^12^/L | Mean | 7.83 | 7.98 | 7.95 | 7.61 | 7.43 | 7.46 | 7.32 | 7.49 | 7.16 | 5.52* | 7.58 | 7.47 |
|  |  | SD | 0.24 | 0.52 | 0.51 | 0.32 | 0.57 | 0.41 | 0.10 | 0.54 | NA | 2.76 | 0.51 | 0.36 |
| **HGB** | g/L | Mean | 152 | 154 | 152 | 148 | 147 | 147 | 145 | 152 | 141 | 113* | 150 | 148 |
|  |  | SD | 4 | 9 | 7 | 5 | 3 | 2 | 5 | 10 | NA | 51 | 6 | 9 |
| **HCT** | % | Mean | 45.40 | 44.4 | 46 | 43.6 | 44.20 | 43.1 | 43.80 | 46.3 | 42.90 | 35.9 | 45.80 | 44.5 |
|  |  | SD | 1.80 | 2.4 | 2.80 | 2.7 | 1.30 | 0.5 | 1.60 | 2.3 | NA | 11.7 | 1.70 | 2.4 |
| **MCV** | fL | Mean | 57.90 | 55.7 | 57.90 | 57.3 | 59.70 | 57.9 | 59.80 | 62 | 59.90 | 71.9* | 60.70 | 59.6 |
|  |  | SD | 1.40 | 1.7 | 2 | 2.8 | 3.40 | 2.9 | 2 | 2.4 | NA | 20.8 | 4.60 | 1.2 |
| **MCH** | pg | Mean | 19.40 | 19.3 | 19.20 | 19.5 | 19.90 | 19.7 | 19.80 | 20.3 | 19.70 | 21.0* | 19.90 | 19.8 |
|  |  | SD | 0.50 | 0.7 | 0.30 | 0.4 | 1.10 | 0.9 | 0.70 | 0.4 | NA | 2 | 1.20 | 0.3 |
| **MCHC** | g/L | Mean | 335 | 346 | 331 | 340 | 332 | 341 | 330 | 329 | 330 | 303* | 3286 | 331 |
|  |  | SD | 9 | 5 | 9 | 11 | 6 | 8 | 3 | 10 | NA | 53 | 6 | 5 |
| **RET#** | 10^9^/L | Mean | 439.5 | 389.3 | 445.1 | 4.78 | 688.0 | 539.5 | 560.1 | 686.3 | 611.2 | 955.9* | 572.7 | 363.8 |
|  |  | SD | 50.7 | 89.5 | 57.6 | 0.36 | 149.5 | 218.7 | 69.4 | 118 | NA | 777.2 | 74.5 | 29.1 |
| **Ret%** | % | Mean | 5.61 | 4.87 | 5.60 | 363.6 | 9.20 | 7.21 | 7.65 | 9.19 | 8.57 | 31.04 | 7.54 | 4.89 |
|  |  | SD | 0.54 | 1 | 0.62 | 25.6 | 1.45 | 2.82 | 0.92 | 1.62 | NA | 41.22 | 0.71 | 0.57 |
| **PLT** | 10^9^/L | Mean | 1168 | 947 | 979 | 1048 | 1106 | 1242 | 1134 | 1137 | 1058 | 817 | 918 | 1314 |
|  |  | SD | 63 | 210 | 210 | 99 | 148 | 333 | 82 | 106 | NA | 309 | 157 | 229 |
| **WBC** | 10^9^/L | Mean | 10.59 | 10.5 | 8.05 | 10.09 | 10.88 | 9.31 | 7.52 | 6.15 | 5.18 | 13.41 | 9.72 | 9.24 |
|  |  | SD | 3.07 | 3.38 | 3.91 | 3.35 | 4.95 | 4.73 | 3.49 | 4.16 | NA | 12.96 | 2.76 | 2.49 |
| **NEUT#** | 10^9^/L | Mean | 1.25 | 0.99 | 0.75 | 1 | 2.27 | 1.32 | 1.45 | 0.76 | 1.42 | 5.66 | 1.29 | 1.06 |
|  |  | SD | 0.57 | 0.33 | 0.34 | 0.36 | 1.70 | 0.92 | 0.50 | 0.57 | NA | 8.12 | 0.40 | 1.02 |
| **NEUT%** | % | Mean | 12.10 | 9.7 | 9.60 | 9.9 | 19.20 | 15.2 | 21.20# | 14 | 27.40 | 28.4 | 13.60 | 11 |
|  |  | SD | 5.30 | 2 | 2.60 | 1.1 | 5.20 | 7.3 | 8.20 | 9.1 | NA | 21.66 | 4.20 | 9.9 |
| **LYMPH#** | 10^9^/L | Mean | 8.81 | 9 | 6.95 | 8.64 | 7.52 | 7.24 | 5.46 | 4.91 | 3.37 | 6.87 | 7.96 | 7.64 |
|  |  | SD | 2.83 | 2.94 | 3.43 | 2.85 | 2.85 | 4.11 | 2.91 | 3.45 | NA | 4.13 | 2.38 | 2.09 |
| **LYMPH%** | % | Mean | 83 | 85.6 | 85.80 | 85.6 | 70.80*## | 76.7 | 70.20*# | 77.1 | 65.00 | 64.3* | 81.70 | 83.4 |
|  |  | SD | 5.70 | 1.9 | 3.30 | 1.6 | 5 | 9.5 | 10.90 | 11.7 | NA | 20.47 | 4.30 | 9.4 |
| **MONO#** | 10^9^/L | Mean | 0.44 | 0.37 | 0.31 | 0.35 | 0.76 | 0.49 | 0.41# | 0.37 | 0.29 | 0.73 | 0.41 | 0.45 |
|  |  | SD | 0.15 | 0.14 | 0.11 | 0.13 | 0.30 | 0.24 | 0.14 | 0.13 | NA | 0.59 | 0.20 | 0.21 |
| **MONO%** | % | Mean | 4.10 | 3.5 | 4 | 3.6 | 7.30 | 5.5 | 5.90## | 7.1**# | 5.60 | 6.1 | 4.10 | 4.7 |
|  |  | SD | 0.70 | 0.5 | 0.80 | 0.8 | 2 | 1.1 | 2.40 | 2.4 | NA | 0.9 | 1.30 | 1.5 |
| **EOS#** | 10^9^/L | Mean | 0.07 | 0.12 | 0.04 | 0.08 | 0.30 | 0.25 | 0.20 | 0.11 | 0.10 | 0.13 | 0.05 | 0.07 |
|  |  | SD | 0.06 | 0.08 | 0.04 | 0.05 | 0.28 | 0.19 | 0.12 | 0.09 | NA | 0.13 | 0.03 | 0.03 |
| **EOS%** | % | Mean | 0.60 | 1.1 | 0.40 | 0.8 | 2.40*# | 2.5*# | 2.60*# | 1.8 | 1.90 | 1 | 0.50 | 0.7 |
|  |  | SD | 0.40 | 0.5 | 0.40 | 0.3 | 1.80 | 1.2 | 0.60 | 1 | NA | 0.5 | 0.30 | 0.3 |
| **BASO#** | 10^9^/L | Mean | 0.02 | 0.01 | 0.01 | 0.02 | 0.02 | 0.02 | 0.01 | 0.01 | 0.01 | 0.01 | 0.02 | 0.02 |
|  |  | SD | 0.02 | 0.01 | 0.01 | 0.01 | 0.02 | 0.02 | 0.01 | 0.01 | NA | 0.01 | 0.03 | 0.01 |
| **BASO%** | % | Mean | 0.10 | 0.1 | 0.10 | 0.2 | 0.20# | 0.2 | 0.10 | 0.1 | 0.20 | 0.2 | 0.10 | 0.3 |
|  |  | SD | 0.20 | 0.1 | 0.10 | 0.1 | 0.10 | 0.2 | 0.10 | 0.1 | NA | 0.2 | 0.20 | 0.1 |

**BASO**, basophil; **EOS**, eosinophil; **HCT**, hematocrit; **HGB**, hemoglobin; **LYMPH**, lymphocyte; **MCH**, Mean corpuscular hemoglobin; **MCHC**, mean corpuscular hemoglobin concentration; **MCV**, mean corpuscular volume; **MONO**, monocyte; **NA**,not available; **NEUT**, neutrophil; **PLT**, platelet; **PM**, polymeric micellar; **RBC**,red blood cell; **RET**,reticulocyte;**WBC**,white blood cell.

a.the concentration of PM was calculated by Methoxy polyethylene glycol-polylactic acid block copolymer

The inter-group differences were assessed using the One-Sample Kolmogorov-Smirnov Test/ Levene’s/ANOVA-Dunnett’s/Dunnett's T3/Mann-Whitney Test.*p≤0.05,compared with 0.9% normal saline group;**, p≤0.01,compared with 0.9% normal saline group;#,p≤0.05,compared with PM group;##,p≤0.01,compared with PM group.

**Supplementary Table 2.** **ZSYY001-induced changes of blood biochemical and coagulogram indexes** **in short-term toxicity test of healthy SD rats(Day 15).**

| **Index** | **Units** | | **0.9% normal saline** | | **PM^a^**  **1000mg/kg** | | **ZSYY001**  **50mg/kg** | | **ZSYY001**  **100mg/kg** | | **ZSYY001**  **200mg/kg** | | **Paclitaxel**  **8mg/kg** | |
| --- | --- | --- | --- | --- | --- | --- | --- | --- | --- | --- | --- | --- | --- | --- |
|  |  |  | **Male**  **(n=5)** | **Female**  **(n=5)** | **Male**  **(n=5)** | **Female**  **(n=5)** | **Male**  **(n=5)** | **Female**  **(n=5)** | **Male**  **(n=5)** | **Female**  **(n=5)** | **Male**  **(n=5)** | **Female**  **(n=5)** | **Male**  **(n=5)** | **Female**  **(n=5)** |
| **TBIL** | mg/dL | Mean | 0.09 | 0.12 | 0.09 | 0.14 | 0.09 | 0.14 | 0.07 | 0.12 | 0.05 | 0.12 | 0.1 | 0.14 |
|  |  | SD | 0.03 | 0.04 | 0.02 | 0.03 | 0.04 | 0.02 | 0.05 | 0.03 | 0.06 | 0.03 | 0.03 | 0.02 |
| **DBIL** | mg/dL | Mean | 0.03 | 0.04 | 0.03 | 0.03 | 0.03 | 0.04 | 0.03 | 0.03 | 0.04 | 0.03 | 0.03 | 0.04 |
|  |  | SD | 0.01 | 0.01 | 0 | 0 | 0.02 | 0 | 0.01 | 0.01 | 0.01 | 0.01 | 0 | 0.01 |
| **ALT** | U/L | Mean | 35 | 32 | 40 | 33 | 42 | 30 | 38 | 35 | 55 | 39 | 35 | 30 |
|  |  | SD | 5 | 6 | 5 | 6 | 9 | 5 | 3 | 6 | 11 | 16 | 4 | 3 |
| **AST** | U/L | Mean | 111 | 138 | 122 | 141 | 117 | 107 | 141 | 187 | 177 | 130 | 114 | 104 |
|  |  | SD | 4 | 37 | 19 | 52 | 17 | 24 | 28 | 124 | 1 | 11 | 41 | 18 |
| **ALP** | U/L | Mean | 238 | 112 | 239 | 171 | 229 | 138 | 177 | 212^**^ | 130 | 121 | 231 | 125 |
|  |  | SD | 47 | 41 | 68 | 54 | 35 | 23 | 67 | 32 | 37 | 29 | 29 | 21 |
| **TP** | g/dL | Mean | 6.3 | 6.8 | 6.2 | 6.8 | 6.3 | 7 | 6.3 | 6.6 | 6.7 | 6.7 | 6.1 | 6.7 |
|  |  | SD | 0.3 | 0.2 | 0.1 | 0.2 | 0.2 | 0.5 | 0.2 | 0.5 | 0.1 | 0.5 | 0.2 | 0.1 |
| **ALB** | g/dL | Mean | 3.3 | 3.8 | 3.3 | 3.8 | 3.3 | 3.9 | 3.3 | 3.6 | 3.3 | 3.6 | 3.2 | 3.7 |
|  |  | SD | 0.1 | 0.2 | 0.2 | 0.1 | 0.1 | 0.4 | 0 | 0.3 | 0.1 | 0.4 | 0.1 | 0.1 |
| **GLB** | g/dL | Mean | 3 | 3 | 2.9 | 3 | 2.9 | 3.1 | 3.1 | 3.1 | 3.4 | 3.1 | 2.9 | 3 |
|  |  | SD | 0.2 | 0.1 | 0.1 | 0.2 | 0.1 | 0.2 | 0.2 | 0.2 | 0.2 | 0.2 | 0.1 | 0.2 |
| **A/G** | N/A | Mean | 1.1 | 1.3 | 1.1 | 1.2 | 1.1 | 1.2 | 1.1 | 1.2 | 1 | 1.2 | 1.1 | 1.2 |
|  |  | SD | 0.1 | 0.1 | 0.1 | 0 | 0 | 0.1 | 0.1 | 0.1 | 0.1 | 0.1 | 0.1 | 0.1 |
| **GLU** | mg/dL | Mean | 138 | 130 | 186 | 171 | 170 | 163 | 165 | 147 | 255 | 228 | 158 | 163 |
|  |  | SD | 36 | 21 | 50 | 59 | 44 | 49 | 36 | 105 | 49 | 37 | 30 | 28 |
| **UREA** | mg/dL | Mean | 28 | 33 | 29 | 39 | 33^**^ | 37 | 29 | 38 | 37 | 44 | 31 | 38 |
|  |  | SD | 2 | 6 | 2 | 8 | 4 | 6 | 2 | 3 | 3 | 5 | 2 | 8 |
| **CREA** | mg/dL | Mean | 0.5 | 0.6 | 0.5 | 0.6 | 0.5 | 0.6 | 0.5 | 0.5 | 0.5 | 0.5 | 0.5 | 0.5 |
|  |  | SD | 0 | 0.1 | 0 | 0.1 | 0 | 0 | 0 | 0.1 | 0 | 0.1 | 0 | 0.1 |
| **TG** | mg/dL | Mean | 35 | 32 | 37 | 28 | 30 | 28 | 36 | 37 | 49 | 51^#^ | 33 | 28 |
|  |  | SD | 8 | 10 | 15 | 6 | 3 | 3 | 11 | 17 | 8 | 10 | 2 | 7 |
| **CHOL** | mg/dL | Mean | 68 | 62 | 65 | 58 | 59 | 64 | 68 | 77^#^ | 77 | 87^##^ | 61 | 74 |
|  |  | SD | 10 | 23 | 11 | 11 | 6 | 9 | 9 | 10 | 10 | 17 | 19 | 29 |
| **CK** | U/L | Mean | 155 | 348 | 218 | 315 | 175 | 188 | 339 | 693 | 377 | 273 | 303 | 191 |
|  |  | SD | 16 | 246 | 111 | 222 | 82 | 72 | 175 | 775 | 113 | 126 | 347 | 84 |
| **NA** | mEq/L | Mean | 148 | 145 | 147 | 146 | 149 | 147 | 149 | 147 | 148 | 147 | 149 | 147 |
|  |  | SD | 2 | 2 | 2 | 2 | 1 | 2 | 2 | 3 | 1 | 0 | 1 | 2 |
| **K** | mEq/L | Mean | 6.7 | 6.6 | 7.1 | 7 | 6.6 | 7.5 | 7.4 | 7.9 | 7.5 | 6.9 | 6.8 | 7.6 |
|  |  | SD | 0.7 | 0.8 | 1.3 | 1.1 | 0.5 | 1.9 | 0.8 | 2.2 | 1.6 | 0.5 | 0.3 | 1.3 |
| **CL** | mEq/L | Mean | 103 | 103 | 104 | 102 | 103 | 103 | 104 | 105 | 104 | 107^#^ | 104 | 104 |
|  |  | SD | 1 | 2 | 1 | 3 | 2 | 2 | 2 | 3 | 1 | 2 | 1 | 2 |
| **CA** | mg/dL | Mean | 11.1 | 11.7 | 11.1 | 11.4 | 10.9 | 11.7 | 11 | 11.3 | 11.3 | 11.7 | 11.1 | 11.5 |
|  |  | SD | 0.3 | 0.5 | 0.2 | 0.3 | 0.6 | 0.5 | 0.3 | 1.1 | 0.4 | 0.9 | 0.2 | 0.3 |
| **PO4** | mg/dL | Mean | 11.2 | 11.8 | 11.2 | 11.5 | 10.7 | 11.2 | 11.9 | 12.4 | 12.2 | 11.7 | 11.3 | 11.8 |
|  |  | SD | 0.8 | 2.4 | 1 | 2.1 | 0.9 | 1.9 | 1 | 2 | 1.1 | 0.3 | 1.1 | 1.8 |
| **PT** | sec | Mean | 12.9 | 12.4 | 13.7 | 11.9 | 13.2 | 12.0 | 12.3 | 10.1 | 12.4 | 7.9 | 12.3 | 10.3 |
|  |  | SD | 1.9 | 1.3 | 0.8 | 2.2 | 1.1 | 1.6 | 1.4 | 2.6 | NA | 2.8 | 2.0 | 1.4 |
| **APTT** | sec | Mean | 9.3 | 8.5 | 9.1 | 8.2 | 9.3 | 8.3 | 9.3 | 8.4 | 9.0 | 8.6 | 9.2 | 8.3 |
|  |  | SD | 0.3 | 0.4 | 0.4 | 0.3 | 0.2 | 0.2 | 0.3 | 0.2 | NA | 0.1 | 0.3 | 0.2 |

**ALB**, albumin; **ALP**, alkaline phosphatase; **ALT**, alanine aminotransferase;**APTT**, activated partial thromboplastin time; **AST**, aspartic transaminase;**CHOL**, cholesterol; **CK**, creatine kinase ; **CREA**, creatinine; **DBIL**, direct bilirubin; **GLB**, globulin; **GLU**,glucose;**PT**, prothrombin time;**TBIL**, total bilirubin;TG, triglyceride; **TP**,total protein;**UREA**,urea.

a.the concentration of PM was calculated by Methoxy polyethylene glycol-polylactic acid block copolymer

The inter-group differences were assessed using the One-Sample Kolmogorov-Smirnov Test/ Levene’s/ANOVA-Dunnett’s/Dunnett's T3/Mann-Whitney Test.**p*≤0.05,compared with 0.9% normal saline group;**,*p*≤0.01,compared with 0.9% normal saline group; #,*p*≤0.05,compared with PM group；##,*p*≤0.01，compared with PM group.

**Supplementary Table 3.** **ZSYY001-induced hemopoietic toxicity in short-term toxicity test of healthy Beagle dogs(Day 15).**

| **Index** | **Units** | | **0.9% normal saline** | | **PM^a^**  **120mg/kg** | | **ZSYY001**  **6mg/kg** | | **ZSYY001**  **12mg/kg** | | **ZSYY001**  **24mg/kg** | | **Paclitaxel**  **8mg/kg** | |
| --- | --- | --- | --- | --- | --- | --- | --- | --- | --- | --- | --- | --- | --- | --- |
|  |  |  | **Male**  **(n=3)** | **Female**  **(n=3)** | **Male**  **(n=3)** | **Female**  **(n=3)** | **Male**  **(n=3)** | **Female**  **(n=3)** | **Male**  **(n=3)** | **Female**  **(n=3)** | **Male**  **(n=2)** | **Female**  **(n=3)** | **Male**  **(n=2)** | **Female**  **(n=2)** |
| **RBC** | 10^12^/L | Mean | 6.75 | 7.04 | 6.52 | 6.83 | 6.45 | 6.18 | 6.63 | 6.54 | 6.34 | 6.61 | 6.83 | 5.28^**^ |
|  |  | SD | 0.20 | 0.21 | 0.44 | 0.67 | 0.30 | 0.36 | 0.57 | 0.45 | NA | 0.32 | NA | NA |
| **HGB** | g/L | Mean | 149 | 163 | 153 | 152 | 144 | 141 | 149 | 147 | 144 | 151 | 145 | 122^**^ |
|  |  | SD | 6 | 8 | 9 | 11 | 5 | 12 | 18 | 11 | NA | 6 | NA | NA |
| **HCT** | % | Mean | 42.2 | 45.4 | 44.1 | 43.8 | 41.7 | 40.0 | 42.7 | 42.1 | 41.4 | 42.9 | 41.3 | 35.0^**^ |
|  |  | SD | 2.8 | 1.1 | 2.8 | 3.5 | 1.2 | 2.2 | 4.8 | 3.2 | NA | 1.7 | NA | NA |
| **MCV** | fL | Mean | 62.6 | 64.5 | 67.6 | 64.1 | 64.7 | 64.7 | 64.3 | 64.3 | 65.2 | 64.8 | 60.6 | 66.3 |
|  |  | SD | 5.3 | 0.4 | 1.3 | 1.1 | 2.2 | 0.8 | 4.3 | 1.9 | NA | 2.1 | NA | NA |
| **MCH** | pg | Mean | 22.1 | 23.1 | 23.5 | 22.3 | 22.4 | 22.8 | 22.4 | 22.4 | 22.7 | 22.9 | 21.2 | 23.0 |
|  |  | SD | 1.3 | 0.5 | 0.5 | 0.6 | 0.6 | 0.7 | 1.6 | 0.6 | NA | 0.3 | NA | NA |
| **MCHC** | g/L | Mean | 353 | 358 | 348 | 348 | 346 | 353 | 349 | 349 | 349 | 353 | 351 | 348 |
|  |  | SD | 10 | 9 | 2 | 4 | 2 | 10 | 5 | 2 | NA | 6 | NA | NA |
| **RET#** | 10^9^/L | Mean | 206.0 | 115.5 | 145.0 | 142.4 | 178.6 | 102.9 | 168.8 | 123.0 | 129.3 | 127.3 | 182.0 | 101.4 |
|  |  | SD | 63.7 | 19.7 | 35.2 | 73.6 | 32.2 | 22.4 | 29.4 | 52.1 | NA | 90.0 | NA | NA |
| **Ret%** | % | Mean | 3.1 | 1.6 | 2.2 | 2.0 | 2.8 | 1.7 | 2.5 | 1.9 | 2.0 | 1.96 | 2.66 | 1.9 |
|  |  | SD | 1.0 | 0.3 | 0.5 | 1.0 | 0.6 | 0.4 | 0.2 | 0.8 | NA | 1.46 | NA | NA |
| **PLT** | 10^9^/L | Mean | 372 | 391 | 318 | 434 | 394 | 556 | 383 | 420 | 317 | 416 | 420 | 540 |
|  |  | SD | 122 | 79 | 45 | 98 | 71 | 125 | 34 | 109 | NA | 51 | NA | NA |
| **WBC** | 10^9^/L | Mean | 10.66 | 11.89 | 10.04 | 14.76 | 14.22 | 11.89 | 12.84 | 11.47 | 13.55 | 9.59 | 8.76 | 9.94 |
|  |  | SD | 2.88 | 3.23 | 2.16 | 2.32 | 0.11 | 2.82 | 2.47 | 2.05 | NA | 11.70 | NA | NA |
| **NEUT#** | 10^9^/L | Mean | 5.18 | 6.57 | 5.55 | 9.33 | 8.74 | 6.92 | 6.63 | 6.85 | 8.62 | 5.48 | 4.50 | 5.96 |
|  |  | SD | 1.74 | 2.08 | 1.31 | 3.40 | 0.62 | 3.11 | 1.90 | 2.33 | NA | 1.62 | NA | NA |
| **NEUT%** | % | Mean | 48.1 | 54.9 | 55.2 | 62.0 | 61.3 | 56.3 | 51.2 | 58.6 | 60.9 | 56.4 | 49.4 | 60.0 |
|  |  | SD | 4.0 | 4.0 | 1.4 | 12.4 | 4.0 | 11.9 | 5.4 | 9.0 | NA | 8.5 | NA | NA |
| **LYMPH#** | 10^9^/L | Mean | 4.5 | 4.6 | 3.9 | 4.6 | 4.3 | 3.9 | 4.8 | 3.9 | 3.4 | 3.28 | 3.2 | 2.8 |
|  |  | SD | 1.0 | 1.0 | 0.8 | 1.4 | 0.4 | 0.2 | 0.9 | 0.1 | NA | 0.44 | NA | NA |
| **LYMPH%** | % | Mean | 43.0 | 38.7 | 38.9 | 32.9 | 30.5 | 33.3 | 37.6 | 34.6 | 28.0 | 34.4 | 37.7 | 28.1 |
|  |  | SD | 6.1 | 4.2 | 0.9 | 13.6 | 3.3 | 5.8 | 1.6 | 6.2 | NA | 2.3 | NA | NA |
| **MONO#** | 10^9^/L | Mean | 0.36 | 0.33 | 0.14 | 0.35 | 0.51 | 0.68 | 0.78 | 0.20 | 1.04^#^ | 0.42 | 0.76 | 0.86 |
|  |  | SD | 0.24 | 0.14 | 0.05 | 0.31 | 0.32 | 0.50 | 0.27 | 0.14 | NA | 0.53 | NA | NA |
| **MONO%** | % | Mean | 3.5 | 2.8 | 1.4 | 2.2 | 3.6 | 6.3 | 6.4^#^ | 1.9 | 7.6^#^ | 4.9 | 9.2^#^ | 8.7 |
|  |  | SD | 2.8 | 1.0 | 0.3 | 1.6 | 2.3 | 4.8 | 2.9 | 1.5 | NA | 6.6 | NA | NA |
| **EOS#** | 10^9^/L | Mean | 0.56 | 0.40 | 0.41 | 0.41 | 0.60 | 0.40 | 0.55 | 0.50 | 0.51 | 0.36 | 0.27 | 0.31 |
|  |  | SD | 0.34 | 0.17 | 0.19 | 0.05 | 0.38 | 0.18 | 0.10 | 0.21 | NA | 0.11 | NA | NA |
| **EOS%** | % | Mean | 4.9 | 3.3 | 4.2 | 2.8 | 4.2 | 3.7 | 4.5 | 4.6 | 3.2 | 3.8 | 3.3 | 3.1 |
|  |  | SD | 1.6 | 1.2 | 2.0 | 0.4 | 2.7 | 2.3 | 1.4 | 2.3 | NA | 0.5 | NA | NA |
| **BASO#** | 10^9^/L | Mean | 0.04 | 0.03 | 0.04 | 0.02 | 0.04 | 0.04 | 0.05 | 0.03 | 0.04 | 0.05 | 0.04 | 0.02 |
|  |  | SD | 0.02 | 0.01 | 0.01 | 0.01 | 0.01 | 0.01 | 0.01 | 0.01 | NA | 0.02 | NA | NA |
| **BASO%** | % | Mean | 0.4 | 0.2 | 0.4 | 0.2 | 0.3 | 0.4 | 0.4 | 0.6 | 0.4 | 0.6 | 0.5 | 0.2 |
|  |  | SD | 0.3 | 0.1 | 0.2 | 0.1 | 0.1 | 0.1 | 0.1 | 0.1 | NA | 0.2 | NA | NA |

**BASO**, basophil; **EOS**, eosinophil; **HCT**, hematocrit; **HGB**, hemoglobin; **LYMPH**, lymphocyte; **MCH**, Mean corpuscular hemoglobin; **MCHC**, mean corpuscular hemoglobin concentration; **MCV**, mean corpuscular volume; **MONO**, monocyte; **NA**,not available; **NEUT**, neutrophil; **PLT**, platelet; **PM**, polymeric micellar; **RBC**,red blood cell; **RET**,reticulocyte;**WBC**,white blood cell.

a.the concentration of PM was calculated by Methoxy polyethylene glycol-polylactic acid block copolymer

The inter-group differences were assessed using the One-Sample Kolmogorov-Smirnov Test/ Levene’s/ANOVA-Dunnett’s/Dunnett's T3/Mann-Whitney Test.**p*≤0.05,compared with 0.9% normal saline group; **,*p*≤0.01,compared with 0.9% normal saline group;#,*p*≤0.05,compared with PM group;##,*p*≤0.01,compared with PM group.

**Supplementary Table 4.** **ZSYY001-induced changes of blood biochemical and coagulogram indexes in short-term toxicity test of healthy Beagle dogs(Day 15).**

| **Index** | **Units** | | **0.9% normal saline** | | **PM^a^**  **120mg/kg** | | **ZSYY001**  **6mg/kg** | | **ZSYY001**  **12mg/kg** | | **ZSYY001**  **24mg/kg** | | **Paclitaxel**  **8mg/kg** | |
| --- | --- | --- | --- | --- | --- | --- | --- | --- | --- | --- | --- | --- | --- | --- |
|  |  |  | **Male**  **(n=3)** | **Female**  **(n=3)** | **Male**  **(n=3)** | **Female**  **(n=3)** | **Male**  **(n=3)** | **Female**  **(n=3)** | **Male**  **(n=3)** | **Female**  **(n=3)** | **Male**  **(n=2)** | **Female**  **(n=3)** | **Male**  **(n=2)** | **Female**  **(n=2)** |
| **TBIL** | mg/dL | Mean | 0.20 | 0.22 | 0.19 | 0.23 | 0.22 | 0.25 | 0.18 | 0.20 | 0.18 | 0.24 | 0.18 | 0.17 |
|  |  | SD | 0.03 | 0.05 | 0.03 | 0.03 | 0.04 | 0.05 | 0.01 | 0.03 | NA | 0.08 | NA | NA |
| **DBIL** | mg/dL | Mean | 0.03 | 0.04 | 0.03 | 0.03 | 0.02 | 0.04 | 0.02 | 0.04 | 0.03 | 0.04 | 0.03 | 0.03 |
|  |  | SD | 0.01 | 0.00 | 0.01 | 0.01 | 0.01 | 0.01 | 0.01 | 0.01 | NA | 0.01 | NA | NA |
| **ALT** | U/L | Mean | 31 | 30 | 35 | 28 | 37 | 30 | 43 | 32 | 35 | 37 | 77 | 75 |
|  |  | SD | 1 | 6 | 5 | 5 | 5 | 8 | 12 | 10 | NA | 3 | NA | NA |
| **AST** | U/L | Mean | 34 | 36 | 35 | 36 | 37 | 40 | 41 | 35 | 34 | 35 | 36 | 30 |
|  |  | SD | 2 | 9 | 4 | 6 | 2 | 8 | 11 | 5 | NA | 7 | NA | NA |
| **ALP** | U/L | Mean | 68 | 88 | 98 | 96 | 90 | 90 | 103 | 53 | 103 | 146 | 142 | 98 |
|  |  | SD | 25 | 44 | 19 | 29 | 25 | 46 | 33 | 12 | NA | 126 | NA | NA |
| **TP** | g/dL | Mean | 6.1 | 6.2 | 5.9 | 6.2 | 6.1 | 6.8 | 6.5^#^ | 6.2 | 6.3 | 6.9 | 6.1 | 5.4 |
|  |  | SD | 0.2 | 0.3 | 0.3 | 0.2 | 0.2 | 1.0 | 0.3 | 0.2 | NA | 0.2 | NA | NA |
| **ALB** | g/dL | Mean | 3.2 | 3.2 | 3.2 | 3.3 | 3.0 | 3.5 | 3.2 | 3.2 | 3.1 | 3.6 | 2.8^*^ | 2.8 |
|  |  | SD | 0.2 | 0.3 | 0.2 | 0.2 | 0.2 | 0.6 | 0.1 | 0.4 | NA | 0.3 | NA | NA |
| **GLB** | g/dL | Mean | 2.9 | 3.0 | 2.7 | 3.0 | 3.0 | 3.0 | 3.3 | 3.0 | 3.2 | 3.3 | 3.4 | 2.6 |
|  |  | SD | 0.2 | 0.1 | 0.1 | 0.1 | 0.2 | 0.4 | 0.3 | 0.2 | NA | 0.3 | NA | NA |
| **A/G** | N/A | Mean | 1.1 | 1.1 | 1.2 | 1.1 | 1.0 | 1.1 | 1.0 | 1.1 | 1.0 | 1.1 | 0.9^*^ | 1.1 |
|  |  | SD | 0.1 | 0.1 | 0.1 | 0.1 | 0.1 | 0.1 | 0.1 | 0.2 | NA | 0.2 | NA | NA |
| **GLU** | mg/dL | Mean | 83 | 84 | 88 | 86 | 90 | 89 | 89 | 94 | 85 | 93 | 88 | 85 |
|  |  | SD | 5 | 5 | 6 | 6 | 6 | 5 | 6 | 7 | NA | 10 | NA | NA |
| **UREA** | mg/dL | Mean | 32 | 30 | 29 | 28 | 31 | 30 | 30 | 24 | 30 | 25 | 24 | 26 |
|  |  | SD | 4 | 1 | 2 | 5 | 5 | 9 | 12 | 3 | NA | 5 | NA | NA |
| **CREA** | mg/dL | Mean | 0.8 | 0.8 | 0.8 | 0.8 | 0.8 | 0.9 | 0.8 | 0.8 | 0.8 | 0.8 | 0.7 | 0.7 |
|  |  | SD | 0.1 | 0.1 | 0.0 | 0.1 | 0.1 | 0.1 | 0.1 | 0.1 | NA | 0.1 | NA | NA |
| **TG** | mg/dL | Mean | 52 | 53 | 44 | 77 | 72 | 58 | 62 | 69 | 59 | 62 | 60 | 54 |
|  |  | SD | 17 | 6 | 8 | 9 | 14 | 7 | 17 | 6 | NA | 15 | NA | NA |
| **CHOL** | mg/dL | Mean | 161 | 188 | 151 | 185 | 195 | 202 | 205 | 180 | 158 | 194 | 186 | 184 |
|  |  | SD | 44 | 48 | 29 | 18 | 41 | 31 | 23 | 25 | NA | 15 | NA | NA |
| **CK** | U/L | Mean | 257 | 267 | 255 | 276 | 230 | 306 | 306 | 231 | 221 | 256 | 218 | 271 |
|  |  | SD | 106 | 115 | 72 | 93 | 56 | 78 | 120 | 62 | NA | 134 | NA | NA |
| **NA** | mEq/L | Mean | 143 | 138 | 141 | 144 | 143 | 152 | 146 | 147 | 141 | 153 | 145 | 133 |
|  |  | SD | 6 | 3 | 6 | 2 | 2 | 12 | 4 | 5 | NA | 3 | NA | NA |
| **K** | mEq/L | Mean | 4.6 | 4.8 | 4.5 | 4.9 | 4.6 | 5.3 | 4.9 | 4.8 | 4.5 | 4.8 | 4.7 | 4.4 |
|  |  | SD | 0.1 | 0.4 | 0.1 | 0.5 | 0.2 | 0.6 | 0.3 | 0.3 | NA | 0.3 | NA | NA |
| **CL** | mEq/L | Mean | 112 | 110 | 112 | 114 | 111 | 116 | 113 | 114 | 111 | 117 | 113 | 103 |
|  |  | SD | 2 | 1 | 4 | 2 | 1 | 8 | 3 | 3 | NA | 2 | NA | NA |
| **CA** | mg/dL | Mean | 10.5 | 10.2 | 10.3 | 10.6 | 10.6 | 11.1 | 10.9 | 10.5 | 10.4 | 11.3 | 10.2 | 9.3 |
|  |  | SD | 0.5 | 0.5 | 0.5 | 0.3 | 0.3 | 1.2 | 0.5 | 0.9 | NA | 0.4 | NA | NA |
| **PO4** | mg/dL | Mean | 5.5 | 4.9 | 5.6 | 5.4 | 6.0 | 5.6 | 6.1 | 4.9 | 5.9 | 5.6 | 6.1 | 5.5 |
|  |  | SD | 0.5 | 1.0 | 0.5 | 0.6 | 0.3 | 0.4 | 0.3 | 0.5 | NA | 0.1 | NA | NA |
| **PT** | sec | Mean | 7.5 | 7.6 | 7.8 | 7.7 | 7.0 | 8.1 | 7.4 | 8.2 | 7.2 | 8.2 | 7.9 | 7.7 |
|  |  | SD | 1.7 | 0.5 | 1.4 | 0.2 | 0.4 | 0.8 | 0.4 | 0.3 | NA | 0.6 | NA | NA |
| **APTT** | sec | Mean | 6.3 | 6.5 | 6.2 | 6.3 | 6.0 | 6.5 | 6.0 | 6.1 | 6.3 | 6.3 | 6.9 | 5.9 |
|  |  | SD | 0.4 | 0.3 | 0.4 | 0.5 | 0.1 | 0.7 | 0.1 | 0.2 | NA | 0.4 | NA | NA |

**ALB**, albumin; **ALP**, alkaline phosphatase; **ALT**, alanine aminotransferase;**APTT**, activated partial thromboplastin time; **AST**, aspartic transaminase;**CHOL**, cholesterol; **CK**, creatine kinase ; **CREA**, creatinine; **DBIL**, direct bilirubin; **GLB**, globulin; **GLU**,glucose;**PT**, prothrombin time;**TBIL**, total bilirubin;TG, triglyceride; **TP**,total protein;**UREA**,urea.

a.the concentration of PM was calculated by Methoxy polyethylene glycol-polylactic acid block copolymer

The inter-group differences were assessed using the One-Sample Kolmogorov-Smirnov Test/ Levene’s/ANOVA-Dunnett’s/Dunnett's T3/Mann-Whitney Test.**p*≤0.05,compared with 0.9% normal saline group;**,*p*≤0.01,compared with 0.9% normal saline group;#,*p*≤0.05,compared with PM group; ##,*p*≤0.01,compared with PM group.

**Supplementary Table 5.ZSYY001-induced hemopoietic toxicity in long-term toxicity test of healthy SD rats(4 weeks).**

| **Index** | **Units** | | **0.9% normal saline** | | **PM^a^**  **450mg/kg** | | **ZSYY001**  **10mg/kg** | | **ZSYY001**  **20mg/kg** | | **ZSYY001**  **30mg/kg** | | **Paclitaxel**  **4mg/kg** | |
| --- | --- | --- | --- | --- | --- | --- | --- | --- | --- | --- | --- | --- | --- | --- |
|  |  |  | **Male**  **(n=10)** | **Female**  **(n=9)** | **Male**  **(n=10)** | **Female**  **(n=10)** | **Male**  **(n=10)** | **Female**  **(n=9)** | **Male**  **(n=9)** | **Female**  **(n=10)** | **Male**  **(n=10)** | **Female**  **(n=10)** | **Male**  **(n=8)** | **Female**  **(n=8)** |
| **RBC** | 10^12^/L | Mean | 8.56 | 8.29 | 8.64 | 8.57 | 7.84^**##^ | 7.89^##^ | 8.2 | 7.36^##^ | 8.81 | 8.21 | 8.37 | 8.28 |
|  |  | SD | 0.61 | 0.40 | 0.31 | 0.39 | 0.54 | 0.28 | 0.5 | 0.28 | 0.45 | 0.52 | 0.55 | 0.32 |
| **HGB** | g/L | Mean | 161 | 157 | 160 | 158 | 154 | 150^*^ | 155 | 140^##^ | 166 | 153 | 158 | 158 |
|  |  | SD | 8 | 5 | 6 | 7 | 9 | 7 | 8 | 6 | 6 | 8 | 10 | 6 |
| **HCT** | % | Mean | 47.3 | 46.1 | 47.3 | 46.4 | 46.4 | 44.8 | 46.9 | 44.9 | 48.3 | 46.4 | 47.2 | 46.2 |
|  |  | SD | 2.2 | 1.7 | 1.8 | 1.7 | 2.2 | 2.2 | 1.6 | 2.0 | 1.7 | 1.4 | 2.7 | 2.4 |
| **MCV** | fL | Mean | 55.3 | 55.7 | 54.7 | 54.1 | 59.3^**##^ | 56.7^#^ | 57.3^#^ | 58.7^##^ | 55 | 54.8 | 56.4 | 56.1 |
|  |  | SD | 2.3 | 1.8 | 1.6 | 1.3 | 2.1 | 1.8 | 2.4 | 2.4 | 2.9 | 1.8 | 2.2 | 1.1 |
| **MCH** | pg | Mean | 18.8 | 19.0 | 18.6 | 18.5 | 19.7^**##^ | 19.0 | 19.0 | 19.0^#^ | 18.9 | 18.6 | 18.9 | 19.1 |
|  |  | SD | 0.7 | 0.6 | 0.4 | 0.4 | 0.7 | 0.6 | 0.8 | 0.5 | 0.8 | 0.5 | 0.5 | 0.4 |
| **MCHC** | g/L | Mean | 340 | 341 | 339 | 341 | 333^#^ | 335^**##^ | 331^#^ | 324^##^ | 344 | 340 | 336 | 341 |
|  |  | SD | 3 | 6 | 6 | 6 | 5 | 3 | 11 | 8 | 9 | 6 | 7 | 4 |
| **RET#** | 10^9^/L | Mean | 317.6 | 330.8 | 293.8 | 291.5 | 499.3^**##^ | 483.8^**##^ | 551.6^##^ | 564.3^##^ | 347.2 | 464.1^*##^ | 375.6 | 417.0 |
|  |  | SD | 20.9 | 58.9 | 43 | 73.7 | 70.6 | 114.5 | 105.0 | 121.2 | 57.6 | 100.3 | 50.6 | 130.7 |
| **Ret%** | % | Mean | 3.72 | 4.00 | 3.41 | 3.40 | 6.37^**##^ | 6.13^**##^ | 6.71^##^ | 7.65^##^ | 3.93 | 5.66^*##^ | 4.50^**^ | 5.03 |
|  |  | SD | 0.29 | 0.74 | 0.52 | 0.84 | 0.75 | 1.41 | 1.15 | 1.56 | 0.54 | 1.20 | 0.64 | 1.57 |
| **PLT** | 10^9^/L | Mean | 1223 | 1188 | 1182 | 1118 | 1081 | 1127 | 1152 | 1069 | 1126 | 1162 | 1153 | 1130 |
|  |  | SD | 108 | 179 | 191 | 209 | 187 | 102 | 214 | 201 | 193 | 271 | 143 | 281 |
| **WBC** | 10^9^/L | Mean | 7.92 | 7.71 | 10.18 | 8.7 | 5.75^##^ | 6.36 | 8.13 | 6.88^$^ | 6.62^#^ | 5.14^##^ | 7.23 | 7.75 |
|  |  | SD | 3.38 | 2.92 | 3.31 | 2.86 | 2.93 | 2.01 | 2.91 | 1.89 | 2.02 | 2.58 | 3.06 | 1.90 |
| **NEUT#** | 10^9^/L | Mean | 0.9 | 1.02 | 1.12 | 0.90 | 0.55^##^ | 0.49^#^ | 0.22 | 0.31^##^ | 0.64^#^ | 0.67 | 0.74 | 0.94 |
|  |  | SD | 0.28 | 0.94 | 0.57 | 0.30 | 0.24 | 0.20 | 0.11 | 0.37 | 0.47 | 0.31 | 0.25 | 0.21 |
| **NEUT%** | % | Mean | 12.1 | 12.5 | 10.9 | 10.7 | 10.6 | 8.1 | 2.9^#^ | 4.0^##^ | 11.7 | 15.3 | 11 | 12.5 |
|  |  | SD | 2.1 | 7.7 | 3.4 | 3.6 | 4.4 | 3.0 | 1.5 | 3.1 | 11.2 | 7.9 | 2.4 | 2.7 |
| **LYMPH#** | 10^9^/L | Mean | 6.63 | 6.24 | 8.49 | 7.26 | 4.66^##^ | 5.39 | 7.17 | 5.91 | 5.06^#^ | 3.85^##^ | 6.08 | 6.31 |
|  |  | SD | 2.94 | 2.25 | 2.74 | 2.53 | 2.45 | 1.86 | 2.82 | 1.48 | 1.92 | 2.11 | 2.76 | 1.74 |
| **LYMPH%** | % | Mean | 83.3 | 81.8 | 83.7 | 83.2 | 80.9 | 84.0 | 85.9 | 86.3 | 74.6^##^ | 71.8^*##^ | 83.0 | 81.1 |
|  |  | SD | 2.1 | 7.4 | 3.8 | 3.2 | 4.9 | 4.7 | 3.7 | 3.6 | 10.3 | 12.6 | 3.8 | 3.9 |
| **MONO#** | 10^9^/L | Mean | 0.3 | 0.37 | 0.45 | 0.45 | 0.44 | 0.38 | 0.75 | 0.57 | 0.84^**^ | 0.54 | 0.34 | 0.39 |
|  |  | SD | 0.16 | 0.19 | 0.21 | 0.21 | 0.25 | 0.11 | 0.25 | 0.17 | 0.57 | 0.26 | 0.13 | 0.08 |
| **MONO%** | % | Mean | 3.61 | 4.70 | 4.23 | 5.11 | 7.55^*^ | 6.18^*^ | 9.50^#^ | 8.38 | 12.42^**##^ | 11.35^**##^ | 5.09 | 5.13 |
|  |  | SD | 1.18 | 1.35 | 1.11 | 1.80 | 2.32 | 1.28 | 2.64 | 1.76 | 6.72 | 5.92 | 1.87 | 1.04 |
| **EOS#** | 10^9^/L | Mean | 0.07 | 0.06 | 0.09 | 0.07 | 0.05 | 0.08 | 0.11 | 0.06 | 0.06 | 0.07 | 0.06 | 0.08 |
|  |  | SD | 0.05 | 0.03 | 0.06 | 0.04 | 0.04 | 0.03 | 0.05 | 0.02 | 0.04 | 0.06 | 0.03 | 0.03 |
| **EOS%** | % | Mean | 0.71 | 0.79 | 0.84 | 0.84 | 0.78 | 1.33 | 1.40 | 0.98 | 0.91 | 1.23 | 0.73 | 1.05 |
|  |  | SD | 0.48 | 0.23 | 0.55 | 0.39 | 0.44 | 0.76 | 0.46 | 0.44 | 0.55 | 0.76 | 0.47 | 0.40 |
| **BASO#** | 10^9^/L | Mean | 0.03 | 0.02 | 0.03 | 0.02 | 0.02 | 0.02 | 0.02 | 0.03 | 0.02 | 0.01 | 0.02 | 0.02 |
|  |  | SD | 0.02 | 0.01 | 0.02 | 0.01 | 0.01 | 0.01 | 0.01 | 0.01 | 0.01 | 0.01 | 0.01 | 0.01 |
| **BASO%** | % | Mean | 0.29 | 0.23 | 0.29 | 0.20 | 0.24 | 0.39 | 0.29 | 0.39 | 0.31 | 0.26 | 0.21 | 0.29 |
|  |  | SD | 0.19 | 0.17 | 0.14 | 0.09 | 0.16 | 0.23 | 0.06 | 0.17 | 0.18 | 0.20 | 0.24 | 0.25 |

**BASO**, basophil; **EOS**, eosinophil; **HCT**, hematocrit; **HGB**, hemoglobin; **LYMPH**, lymphocyte; **MCH**, Mean corpuscular hemoglobin; **MCHC**, mean corpuscular hemoglobin concentration; **MCV**, mean corpuscular volume; **MONO**, monocyte; **NA**,not available; **NEUT**, neutrophil; **PLT**, platelet; **PM**, polymeric micellar; **RBC**,red blood cell; **RET**,reticulocyte;**WBC**,white blood cell.

a.the concentration of PM was calculated by Methoxy polyethylene glycol-polylactic acid block copolymer

The inter-group differences were assessed using the One-Sample Kolmogorov-Smirnov Test/ Levene’s/ANOVA-Dunnett’s/Dunnett's T3/Mann-Whitney Test.**p*≤0.05,compared with 0.9% normal saline group;**,*p*≤0.01,compared with 0.9% normal saline group;#,*p*≤0.05,compared with PM group; ##,*p*≤0.01,compared with PM group.

**Supplementary Table 6.** **ZSYY001-induced changes of blood biochemical and coagulogram indexes in long-term toxicity test of healthy SD rats(4 weeks).**

| **Index** | **Units** | | **0.9% normal saline** | | **PM^a^**  **450mg/kg** | | **ZSYY001**  **10mg/kg** | | **ZSYY001**  **20mg/kg** | | **ZSYY001**  **30mg/kg** | | **Paclitaxel**  **4mg/kg** | |
| --- | --- | --- | --- | --- | --- | --- | --- | --- | --- | --- | --- | --- | --- | --- |
|  |  |  | **Male**  **(n=10)** | **Female**  **(n=9)** | **Male**  **(n=10)** | **Female**  **(n=10)** | **Male**  **(n=10)** | **Female**  **(n=9)** | **Male**  **(n=9)** | **Female**  **(n=10)** | **Male**  **(n=10)** | **Female**  **(n=10)** | **Male**  **(n=8)** | **Female**  **(n=8)** |
| **TBIL** | mg/dL | Mean | 0.11 | 0.15 | 0.11 | 0.17 | 0.10 | 0.14 | 0.07^##^ | 0.16^#^ | 0.16 | 0.15 | 0.10 | 0.14 |
|  |  | SD | 0.03 | 0.05 | 0.02 | 0.07 | 0.02 | 0.05 | 0.03 | 0.03 | 0.13 | 0.05 | 0.04 | 0.05 |
| **ALT** | U/L | Mean | 38 | 42 | 36 | 37 | 45^#^ | 38 | 43 | 30 | 68^**##^ | 113^#^ | 37 | 37 |
|  |  | SD | 8 | 18 | 8 | 13 | 12 | 15 | 7 | 9 | 34 | 192 | 6 | 7 |
| **AST** | U/L | Mean | 112 | 137 | 115 | 117 | 124 | 110 | 124 | 96 | 297^**##^ | 358^##^ | 113 | 120 |
|  |  | SD | 25 | 92 | 22 | 30 | 43 | 43 | 18 | 20 | 226 | 528 | 37 | 40 |
| **ALP** | U/L | Mean | 197 | 150 | 195 | 128 | 192 | 132 | 148^##^ | 84 | 198 | 105^**^ | 198 | 146 |
|  |  | SD | 37 | 67 | 29 | 61 | 43 | 64 | 33 | 26 | 159 | 47 | 28 | 65 |
| **TP** | g/dL | Mean | 6.6 | 6.9 | 6.5 | 6.9 | 6.4 | 6.7^#^ | 6.2^##^ | 6.4 | 6.4 | 6.7 | 6.4 | 6.8 |
|  |  | SD | 0.3 | 0.4 | 0.4 | 0.7 | 0.3 | 0.5 | 0.4 | 0.2 | 0.6 | 0.7 | 0.3 | 0.6 |
| **ALB** | g/dL | Mean | 3.6 | 3.8 | 3.5 | 3.9 | 3.5 | 3.7^#^ | 3.6^##^ | 3.9^##^ | 3.4 | 3.7 | 3.6 | 3.9 |
|  |  | SD | 0.2 | 0.3 | 0.2 | 0.5 | 0.1 | 0.3 | 0.2 | 0.2 | 0.4 | 0.5 | 0.2 | 0.4 |
| **GLB** | g/dL | Mean | 3.0 | 3.1 | 2.9 | 3.1 | 2.9 | 2.9 | 2.7^##^ | 2.5^##^ | 3.0 | 3.0^#^ | 2.8 | 2.9 |
|  |  | SD | 0.2 | 0.2 | 0.2 | 0.2 | 0.2 | 0.2 | 0.2 | 0.1 | 0.3 | 0.3 | 0.2 | 0.2 |
| **A/G** | N/A | Mean | 1.2 | 1.2 | 1.2 | 1.3 | 1.2 | 1.3 | 1.3 | 1.5^##^ | 1.1^#^ | 1.2 | 1.3 | 1.4 |
|  |  | SD | 0.1 | 0.1 | 0.1 | 0.1 | 0.1 | 0.1 | 0.1 | 0.1 | 0.1 | 0.1 | 0.1 | 0.1 |
| **GLU** | mg/dL | Mean | 161 | 144 | 139 | 133 | 151 | 155^*^ | 169 | 127 | 156 | 172^**^ | 150 | 135 |
|  |  | SD | 48 | 52 | 34 | 32 | 60 | 57 | 58 | 37 | 90 | 58 | 37 | 37 |
| **UREA** | mg/dL | Mean | 32 | 36 | 28^*^ | 37 | 28^*^ | 34 | 32^#^ | 31^#^ | 53^**##^ | 51 | 29 | 37 |
|  |  | SD | 4 | 6 | 3 | 8 | 3 | 5 | 4 | 3 | 27 | 22 | 3 | 9 |
| **CREA** | mg/dL | Mean | 0.6 | 0.6 | 0.5 | 0.6 | 0.5^**^ | 0.5 | 0.5^**^ | 0.5 | 0.5 | 0.5 | 0.5 | 0.6 |
|  |  | SD | 0.0 | 0.0 | 0.1 | 0.1 | 0.0 | 0.1 | 0.0 | 0.1 | 0.1 | 0.1 | 0.1 | 0.1 |
| **TG** | mg/dL | Mean | 46 | 45 | 62^*^ | 49 | 52 | 53 | 31^##^ | 39 | 28^*##^ | 28^**##^ | 53 | 53 |
|  |  | SD | 10 | 9 | 18 | 16 | 8 | 11 | 9 | 9 | 12 | 9 | 17 | 19 |
| **CHOL** | mg/dL | Mean | 56 | 71 | 64 | 75 | 72 | 80 | 64 | 62^##^ | 56 | 57 | 70 | 75 |
|  |  | SD | 14 | 22 | 20 | 22 | 16 | 25 | 10 | 12 | 18 | 15 | 13 | 21 |
| **CK** | U/L | Mean | 199 | 316 | 252 | 274 | 241 | 210 | 237 | 174 | 748 | 450 | 275 | 316 |
|  |  | SD | 70 | 365 | 86 | 166 | 145 | 100 | 90 | 79 | 1261 | 600 | 195 | 217 |
| **NA** | mEq/L | Mean | 148 | 146 | 147 | 145 | 148 | 147 | 145 | 143 | 149 | 148 | 147 | 146 |
|  |  | SD | 3 | 1 | 3 | 2 | 1 | 1 | 3 | 2 | 3 | 3 | 1 | 2 |
| **K** | mEq/L | Mean | 7.2 | 8.0 | 7.1 | 7.3 | 6.7 | 7.1 | 8.5^#^ | 9.2 | 7.3 | 7.4 | 6.5 | 6.6 |
|  |  | SD | 1.6 | 1.4 | 1.6 | 1.6 | 1.0 | 1.2 | 1.5 | 2.0 | 1.1 | 0.9 | 0.8 | 0.8 |
| **CL** | mEq/L | Mean | 103 | 104 | 102 | 104 | 103 | 105 | 105^##^ | 106^##^ | 107^*##^ | 106 | 102 | 103 |
|  |  | SD | 1 | 1 | 2 | 1 | 2 | 1 | 1 | 2 | 3 | 3 | 2 | 2 |
| **CA** | mg/dL | Mean | 11.7 | 11.9 | 11.4 | 11.7 | 11.8^#^ | 11.8 | 11.6 | 11.8 | 11.0 | 11.5 | 11.6 | 11.7 |
|  |  | SD | 0.5 | 0.3 | 0.2 | 0.6 | 0.3 | 0.5 | 0.5 | 0.5 | 1.4 | 0.7 | 0.2 | 0.5 |
| **PO4** | mg/dL | Mean | 11.5 | 11.8 | 11.3 | 10.9 | 11.5 | 11.1 | 13.0 | 11.3 | 11.2 | 11.0 | 11.3 | 10.4 |
|  |  | SD | 0.8 | 0.8 | 1.2 | 1.4 | 1.2 | 1.6 | 1.1 | 0.9 | 1.5 | 1.3 | 0.6 | 1.3 |
| **PT** | sec | Mean | 15.3 | 12.0 | 13.6 | 11.9 | 13.5 | 13.1 | 14.0 | 11.3 | 15.1 | 13.3 | 14.5 | 13.6 |
|  |  | SD | 1.8 | 1.9 | 1.7 | 1.7 | 2.5 | 1.9 | 2.5 | 2.0 | 2.5 | 2.0 | 2.0 | 1.8 |
| **APTT** | sec | Mean | 9.8 | 8.0 | 9.1^**^ | 7.8^*^ | 9.3^*^ | 7.9 | 9.2 | 8.3^##^ | 10.2 | 8.3 | 9.5 | 8.0 |
|  |  | SD | 0.5 | 0.2 | 0.4 | 0.2 | 0.4 | 0.2 | 0.4 | 0.2 | 3.2 | 0.9 | 0.4 | 0.3 |

**ALB**, albumin; **ALP**, alkaline phosphatase; **ALT**, alanine aminotransferase;**APTT**, activated partial thromboplastin time; **AST**, aspartic transaminase;**CHOL**, cholesterol; **CK**, creatine kinase ; **CREA**, creatinine; **DBIL**, direct bilirubin; **GLB**, globulin; **GLU**,glucose;**PT**, prothrombin time;**TBIL**, total bilirubin;TG, triglyceride; **TP**,total protein;**UREA**,urea.

a.the concentration of PM was calculated by Methoxy polyethylene glycol-polylactic acid block copolymer

The inter-group differences were assessed using the One-Sample Kolmogorov-Smirnov Test/ Levene’s/ANOVA-Dunnett’s/Dunnett's T3/Mann-Whitney Test.**p*≤0.05,compared with 0.9% normal saline group;**,*p*≤0.01,compared with 0.9% normal saline group;#,*p*≤0.05,compared with PM group; ##,*p*≤0.01,compared with PM group.

**Supplementary Table 7. ZSYY001-induced hemopoietic toxicity in long-term toxicity test of healthy Beagle dogs(4 weeks).**

| **Index** | **Units** | | **0.9% normal saline** | | **PM^a^**  **60mg/kg** | | **ZSYY001**  **3mg/kg** | | **ZSYY001**  **6mg/kg** | | **ZSYY001**  **12mg/kg** | | **Paclitaxel**  **2mg/kg** | |
| --- | --- | --- | --- | --- | --- | --- | --- | --- | --- | --- | --- | --- | --- | --- |
|  |  |  | **Male**  **(n=5)** | **Female**  **(n=5)** | **Male**  **(n=5)** | **Female**  **(n=5)** | **Male**  **(n=5)** | **Female**  **(n=5)** | **Male**  **(n=5)** | **Female**  **(n=5)** | **Male**  **(n=5)** | **Female**  **(n=5)** | **Male**  **(n=5)** | **Female**  **(n=5)** |
| **RBC** | 10^12^/L | Mean | 6.92 | 7.02 | 7.14 | 6.98 | 6.12^*##^ | 6.58 | 6.11^*##^ | 6.44 | 6.19^##^ | 6.23 | 6.47 | 6.22 |
|  |  | SD | 0.72 | 0.22 | 0.39 | 0.51 | 0.32 | 0.53 | 0.37 | 0.55 | 0.33 | 0.41 | 0.52 | 0.65 |
| **HGB** | g/L | Mean | 154 | 159 | 159 | 155 | 131^*##^ | 142 | 132^*##^ | 142 | 134^*##^ | 139* | 136 | 141 |
|  |  | SD | 15 | 8 | 13 | 10 | 10 | 12 | 9 | 14 | 11 | 7 | 10 | 16 |
| **HCT** | % | Mean | 44.2 | 46.7 | 46.1 | 44.9 | 39.3^##^ | 42.2 | 39.4^##^ | 42.4 | 40.4^#^ | 41.5* | 40.6 | 41.9 |
|  |  | SD | 3.5 | 1.9 | 3.3 | 3.4 | 2.7 | 2.7 | 2.3 | 3.1 | 2.8 | 1.8 | 2.8 | 4.2 |
| **MCV** | fL | Mean | 64.2 | 66.5 | 64.6 | 64.4 | 64.2 | 64.3 | 64.6 | 66.0 | 65.3 | 66.6 | 62.8 | 67.5 |
|  |  | SD | 3.7 | 1.6 | 3.3 | 4.7 | 1.9 | 3.0 | 2.4 | 3.7 | 2.4 | 2.2 | 1.9 | 2.6 |
| **MCH** | pg | Mean | 22.3 | 22.7 | 22.3 | 22.2 | 21.4 | 21.6 | 21.6 | 22.0 | 21.6 | 22.3 | 21.0 | 22.6 |
|  |  | SD | 0.7 | 0.7 | 1.3 | 1.1 | 0.7 | 0.8 | 0.5 | 1.0 | 1.0 | 0.8 | 0.5 | 0.8 |
| **MCHC** | g/L | Mean | 349 | 341 | 345 | 345 | 334^*#^ | 336 | 335^**#^ | 334 | 332^**#^ | 334 | 334^*^ | 336 |
|  |  | SD | 12 | 5 | 6 | 9 | 3 | 10 | 7 | 11 | 8 | 6 | 4 | 6 |
| **RET#** | 10^9^/L | Mean | 156.9 | 111.2 | 127.4 | 142.6 | 175.5 | 171.1 | 143.6 | 178.7 | 108.6 | 128.4 | 177.4 | 143.3 |
|  |  | SD | 45.5 | 46.5 | 53.9 | 69.3 | 104.3 | 93.7 | 38.1 | 46.7 | 55.5 | 45.0 | 52.7 | 78.0 |
| **Ret%** | % | Mean | 2.28 | 1.58 | 1.76 | 2.01 | 2.83 | 2.55 | 2.35 | 2.80 | 1.77 | 2.10 | 2.73 | 2.28 |
|  |  | SD | 0.67 | 0.64 | 0.65 | 0.94 | 1.54 | 1.24 | 0.60 | 0.80 | 0.96 | 0.86 | 0.74 | 1.15 |
| **PLT** | 10^9^/L | Mean | 445 | 373 | 284^*^ | 385 | 380 | 399 | 376 | 448 | 403 | 414 | 382 | 379 |
|  |  | SD | 49 | 77 | 102 | 51 | 127 | 93 | 69 | 138 | 73 | 76 | 71 | 41 |
| **WBC** | 10^9^/L | Mean | 11.42 | 10.33 | 10.87 | 11.66 | 8.33 | 8.49# | 8.12 | 8.31# | 9.47 | 7.55## | 8.95 | 7.22 |
|  |  | SD | 2.48 | 4.07 | 2.63 | 2.55 | 0.91 | 1.26 | 1.81 | 1.87 | 1.07 | 0.99 | 2.31 | 0.23 |
| **NEUT#** | 10^9^/L | Mean | 6.39 | 5.70 | 6.41 | 6.94 | 3.90^#^ | 4.21# | 3.72^#^ | 3.91## | 4.35 | 3.43 | 4.72 | 3.49 |
|  |  | SD | 1.81 | 2.20 | 2.00 | 2.07 | 0.91 | 0.93 | 1.66 | 1.33 | 1.00 | 0.82 | 1.77 | 0.44 |
| **NEUT%** | % | Mean | 55.5 | 55.3 | 58.3 | 58.7 | 46.9 | 49.3 | 44.2^#^ | 46.2# | 45.6 | 45.0*## | 51.5 | 48.3 |
|  |  | SD | 4.8 | 3.5 | 5.7 | 5.0 | 9.8 | 5.9 | 9.6 | 6.2 | 8.3 | 6.7 | 7.8 | 5.4 |
| **LYMPH#** | 10^9^/L | Mean | 4.32 | 3.78 | 3.65 | 3.88 | 3.63 | 3.74 | 3.32 | 3.65 | 3.90 | 3.13 | 3.30 | 3.27 |
|  |  | SD | 0.72 | 1.32 | 0.68 | 0.49 | 0.85 | 0.52 | 0.23 | 0.63 | 0.74 | 0.37 | 0.62 | 0.43 |
| **LYMPH%** | % | Mean | 38.2 | 37.1 | 34.2 | 34.4 | 43.3 | 44.5 | 42.6 | 44.8 | 41.6 | 41.6 | 38.0 | 45.4 |
|  |  | SD | 3.2 | 2.3 | 5.0 | 7.4 | 8.0 | 7.0 | 10.1 | 6.6 | 9.8 | 3.9 | 8.0 | 6.2 |
| **MONO#** | 10^9^/L | Mean | 0.30 | 0.28 | 0.30 | 0.33 | 0.63 | 0.36 | 1.00*# | 0.56 | 1.06**# | 0.87# | 0.75 | 0.30 |
|  |  | SD | 0.24 | 0.19 | 0.27 | 0.33 | 0.37 | 0.28 | 0.40 | 0.23 | 0.45 | 0.28 | 0.19 | 0.14 |
| **MONO%** | % | Mean | 2.7 | 3.2 | 2.7 | 2.8 | 7.7 | 3.9 | 12.2**## | 6.7 | 10.9**## | 11.5**## | 8.4* | 4.2 |
|  |  | SD | 1.9 | 2.6 | 2.3 | 2.5 | 4.4 | 2.7 | 4.4 | 2.0 | 3.5 | 3.5 | 0.3 | 1.9 |
| **EOS#** | 10^9^/L | Mean | 0.35 | 0.54 | 0.44 | 0.47 | 0.17# | 0.13* | 0.08**## | 0.12** | 0.13^*##^ | 0.11 | 0.13* | 0.11** |
|  |  | SD | 0.08 | 0.68 | 0.15 | 0.25 | 0.13 | 0.06 | 0.08 | 0.02 | 0.14 | 0.12 | 0.02 | 0.03 |
| **EOS%** | % | Mean | 3.2 | 4.2 | 4.2 | 3.8 | 2.1 | 1.6 | 1.0# | 1.5 | 1.5# | 1.7 | 1.6 | 1.5 |
|  |  | SD | 0.9 | 3.3 | 1.5 | 1.5 | 1.8 | 0.8 | 1.0 | 0.2 | 1.7 | 1.9 | 0.5 | 0.5 |
| **BASO#** | 10^9^/L | Mean | 0.05 | 0.03 | 0.06 | 0.04 | 0.01 | 0.05 | 0.03 | 0.07 | 0.03 | 0.01 | 0.05 | 0.04 |
|  |  | SD | 0.03 | 0.02 | 0.03 | 0.03 | 0.01 | 0.02 | 0.06 | 0.04 | 0.03 | 0.02 | 0.03 | 0.03 |
| **BASO%** | % | Mean | 0.5 | 0.3 | 0.6 | 0.4 | 0.1 | 0.6 | 0.4 | 0.8 | 0.4 | 0.2 | 0.5 | 0.6 |
|  |  | SD | 0.4 | 0.2 | 0.3 | 0.3 | 0.1 | 0.3 | 0.9 | 0.4 | 0.4 | 0.3 | 0.4 | 0.4 |

**BASO**, basophil; **EOS**, eosinophil; **HCT**, hematocrit; **HGB**, hemoglobin; **LYMPH**, lymphocyte; **MCH**, Mean corpuscular hemoglobin; **MCHC**, mean corpuscular hemoglobin concentration; **MCV**, mean corpuscular volume; **MONO**, monocyte; **NA**,not available; **NEUT**, neutrophil; **PLT**, platelet; **PM**, polymeric micellar; **RBC**,red blood cell; **RET**,reticulocyte;**WBC**,white blood cell.

a.the concentration of PM was calculated by Methoxy polyethylene glycol-polylactic acid block copolymer

The inter-group differences were assessed using the One-Sample Kolmogorov-Smirnov Test/ Levene’s/ANOVA-Dunnett’s/Dunnett's T3/Mann-Whitney Test.**p*≤0.05,compared with 0.9% normal saline group;**,*p*≤0.01,compared with 0.9% normal saline group;#,*p*≤0.05,compared with PM group; ##,*p*≤0.01,compared with PM group.

**Supplementary Table 8.** **ZSYY001-induced changes of blood biochemical and coagulogram indexes in long-term toxicity test of healthy Beagle dogs(4 weeks).**

| **Index** | **Units** | | **0.9% normal saline** | | **PM^a^**  **60mg/kg** | | **ZSYY001**  **3mg/kg** | | **ZSYY001**  **6mg/kg** | | **ZSYY001**  **12mg/kg** | | **Paclitaxel**  **2mg/kg** | |
| --- | --- | --- | --- | --- | --- | --- | --- | --- | --- | --- | --- | --- | --- | --- |
|  |  |  | **Male**  **(n=5)** | **Female**  **(n=5)** | **Male**  **(n=5)** | **Female**  **(n=5)** | **Male**  **(n=5)** | **Female**  **(n=5)** | **Male**  **(n=5)** | **Female**  **(n=5)** | **Male**  **(n=5)** | **Female**  **(n=5)** | **Male**  **(n=5)** | **Female**  **(n=5)** |
| **TBIL** | mg/dL | Mean | 0.03 | 0.03 | 0.03 | 0.03 | 0.02 | 0.02 | 0.02 | 0.02 | 0.02* | 0.02 | 0.02 | 0.02 |
|  |  | SD | 0.01 | 0.01 | 0.01 | 0.01 | 0.01 | 0.01 | 0.01 | 0.01 | 0.00 | 0.01 | 0.01 | 0.01 |
| **ALT** | U/L | Mean | 36 | 29 | 33 | 35 | 64 | 26# | 60 | 31 | 94 | 82 | 31 | 32 |
|  |  | SD | 7 | 7 | 4 | 3 | 75 | 2 | 55 | 6 | 117 | 80 | 6 | 9 |
| **AST** | U/L | Mean | 36 | 34 | 32 | 34 | 32 | 29 | 32 | 32 | 29 | 28 | 30 | 29 |
|  |  | SD | 8 | 7 | 7 | 6 | 5 | 5 | 7 | 10 | 6 | 5 | 4 | 3 |
| **ALP** | U/L | Mean | 87 | 70 | 90 | 88 | 115 | 73 | 121 | 96 | 155# | 145 | 121 | 76 |
|  |  | SD | 38 | 15 | 28 | 25 | 34 | 21 | 36 | 23 | 49 | 77 | 50 | 24 |
| **TP** | g/dL | Mean | 6.4 | 6.2 | 6.3 | 6.3 | 6.1 | 6.1 | 6.1 | 6.2 | 5.9* | 6.0 | 6.4 | 6.4 |
|  |  | SD | 0.2 | 0.3 | 0.4 | 0.2 | 0.2 | 0.2 | 0.2 | 0.3 | 0.2 | 0.4 | 0.2 | 0.2 |
| **ALB** | g/dL | Mean | 3.3 | 3.3 | 3.4 | 3.4 | 3.3 | 3.3 | 3.1 | 3.4 | 3.0*# | 3.2 | 3.5 | 3.6** |
|  |  | SD | 0.2 | 0.1 | 0.2 | 0.2 | 0.3 | 0.2 | 0.1 | 0.2 | 0.2 | 0.2 | 0.1 | 0.1 |
| **GLB** | g/dL | Mean | 3.1 | 2.9 | 2.9 | 2.9 | 2.8 | 2.8 | 3.0 | 2.8 | 2.9 | 2.8 | 3.0 | 2.7 |
|  |  | SD | 0.4 | 0.3 | 0.3 | 0.2 | 0.2 | 0.0 | 0.3 | 0.3 | 0.1 | 0.3 | 0.1 | 0.2 |
| **A/G** | N/A | Mean | 1.1 | 1.2 | 1.2 | 1.2 | 1.1 | 1.2 | 1.1 | 1.2 | 1.0 | 1.1 | 1.2 | 1.4 |
|  |  | SD | 0.2 | 0.1 | 0.1 | 0.1 | 0.2 | 0.1 | 0.1 | 0.2 | 0.1 | 0.1 | 0.1 | 0.1 |
| **GLU** | mg/dL | Mean | 97 | 95 | 96 | 93 | 99 | 87 | 94 | 93 | 94 | 97 | 97 | 95 |
|  |  | SD | 8 | 11 | 3 | 14 | 9 | 7 | 6 | 11 | 10 | 7 | 7 | 7 |
| **UREA** | mg/dL | Mean | 34 | 32 | 29 | 37 | 27 | 32 | 30 | 30 | 29 | 26 | 34 | 34 |
|  |  | SD | 16 | 8 | 9 | 9 | 5 | 3 | 4 | 8 | 9 | 7 | 4 | 4 |
| **CREA** | mg/dL | Mean | 0.8 | 0.8 | 0.8 | 0.9 | 0.8 | 0.8 | 0.7# | 0.8 | 0.7# | 0.7 | 0.8 | 0.8 |
|  |  | SD | 0.1 | 0.0 | 0.1 | 0.1 | 0.0 | 0.1 | 0.1 | 0.1 | 0.0 | 0.0 | 0.1 | 0.1 |
| **TG** | mg/dL | Mean | 58 | 53 | 49 | 56 | 46 | 52 | 66 | 59 | 54 | 57 | 57 | 48 |
|  |  | SD | 13 | 14 | 12 | 6 | 10 | 10 | 16 | 9 | 4 | 6 | 8 | 6 |
| **CHOL** | mg/dL | Mean | 164 | 176 | 151 | 165 | 145 | 175 | 180 | 180 | 175 | 197 | 188 | 185 |
|  |  | SD | 43 | 38 | 19 | 24 | 20 | 40 | 12 | 20 | 21 | 37 | 15 | 42 |
| **CK** | U/L | Mean | 271 | 291 | 158 | 223 | 201 | 203 | 235 | 265 | 218 | 203 | 187 | 171 |
|  |  | SD | 97 | 76 | 46 | 61 | 49 | 97 | 80 | 162 | 76 | 53 | 48 | 42 |
| **NA** | mEq/L | Mean | 146 | 146 | 147 | 147 | 149 | 148 | 150 | 150* | 151## | 150* | 150 | 150* |
|  |  | SD | 3 | 1 | 1 | 2 | 1 | 2 | 1 | 1 | 2 | 1 | 2 | 2 |
| **K** | mEq/L | Mean | 4.8 | 4.7 | 4.4 | 4.6 | 5.1## | 5.0# | 5.0# | 5.2*## | 5.1## | 5.0 | 5.3* | 4.9 |
|  |  | SD | 0.2 | 0.3 | 0.3 | 0.1 | 0.3 | 0.2 | 0.4 | 0.3 | 0.2 | 0.3 | 0.3 | 0.2 |
| **CL** | mEq/L | Mean | 114 | 115 | 114 | 116 | 117*# | 116 | 117*# | 118* | 119**## | 118 | 117* | 118 |
|  |  | SD | 2 | 2 | 1 | 3 | 1 | 2 | 1 | 1 | 2 | 2 | 2 | 2 |
| **CA** | mg/dL | Mean | 10.8 | 10.2 | 10.4 | 10.5** | 10.5 | 10.6** | 10.6 | 10.8**# | 10.6 | 10.7 | 11.0 | 10.9** |
|  |  | SD | 0.4 | 0.1 | 0.2 | 0.1 | 0.3 | 0.2 | 0.1 | 0.2 | 0.4 | 0.4 | 0.2 | 0.1 |
| **PO4** | mg/dL | Mean | 5.7 | 5.1 | 4.7 | 4.9 | 5.6 | 4.6 | 5.0 | 5.5 | 5.5 | 5.5 | 5.7 | 5.4 |
|  |  | SD | 0.8 | 0.4 | 1.2 | 0.4 | 0.6 | 0.4 | 0.5 | 0.7 | 0.5 | 0.8 | 0.5 | 0.7 |
| **PT** | sec | Mean | 7.6 | 6.8 | 7.4 | 7.2 | 7.6 | 7.8 | 7.5 | 8.2* | 8.5 | 8.0* | 7.2 | 7.5 |
|  |  | SD | 0.8 | 0.2 | 0.7 | 0.6 | 0.7 | 0.3 | 0.5 | 1.0 | 1.5 | 0.4 | 1.0 | 1.0 |
| **APTT** | sec | Mean | 6.3 | 6.2 | 5.8 | 5.9 | 6.2 | 5.9 | 6.0 | 5.8 | 6.0 | 5.9 | 5.9 | 5.9 |
|  |  | SD | 0.6 | 0.2 | 0.2 | 0.3 | 0.4 | 0.5 | 0.4 | 0.1 | 0.8 | 0.6 | 0.8 | 0.1 |

**ALB**, albumin; **ALP**, alkaline phosphatase; **ALT**, alanine aminotransferase;**APTT**, activated partial thromboplastin time; **AST**, aspartic transaminase;**CHOL**, cholesterol; **CK**, creatine kinase ; **CREA**, creatinine; **DBIL**, direct bilirubin; **GLB**, globulin; **GLU**,glucose;**PT**, prothrombin time;**TBIL**, total bilirubin;TG, triglyceride; **TP**,total protein;**UREA**,urea.

a.the concentration of PM was calculated by Methoxy polyethylene glycol-polylactic acid block copolymer

The inter-group differences were assessed using the One-Sample Kolmogorov-Smirnov Test/ Levene’s/ANOVA-Dunnett’s/Dunnett's T3/Mann-Whitney Test.**p*≤0.05,compared with 0.9% normal saline group;**,*p*≤0.01,compared with 0.9% normal saline group;#,*p*≤0.05,compared with PM group;##,*p*≤0.01,compared with PM group.

**Supplementary Table 9.** **Pharmacokinetic parameters of free and total paclitaxel in plasma after the first dose(Mean±SD).**

| Dose level | | C_max_(ng/mL) | AUC_0-t_ (ng·h/mL) | AUC_0-∞_ (ng·h/mL) | t_1/2_(h) | T_max_(h) | λz(/h) | Vd(L) | CL(L/h) | MRT_0-t_ (h) | MRT_0-∞_ (h) |
| --- | --- | --- | --- | --- | --- | --- | --- | --- | --- | --- | --- |
| 175 mg/m^2^  (n=3) | Total | 4370.00±1361.95 | 6202.15±2188.11 | 6668.48±2232.88 | 13.19±4.34 | 0.98±0.03 | 0.06±0.02 | 770.10±130.80 | 42.58±12.55 | 4.09±1.36 | 7.37±1.83 |
|  | Free | 131.33±28.68 | 149.89±32.62 | 162.81±38.08 | 5.48±2.54 | 0.82±0.28 | 0.15±0.08 | 12342.57±2323.07 | 1728.19±558.62 | 1.32±0.32 | 2.18±1.27 |
| 230 mg/m^2^  (n=5) | Total | 7866.00±2383.73 | 12207.78±3219.82 | 12626.69±3261.48 | 16.45±4.79 | 0.86±0.24 | 0.05±0.02 | 678.77±132.17 | 29.92±6.44 | 6.14±2.32 | 8.65±2.88 |
|  | Free | 231.60±95.02 | 272.14±82.59 | 288.66±82.95 | 7.44±2.73 | 0.75±0.25 | 0.10±0.04 | 13477.70±3348.33 | 1342.39±385.94 | 1.86±0.95 | 3.33±1.57 |
| 300 mg/m^2^  (n=3) | Total | 8526.67±1895.11 | 14085.18±3394.09 | 14742.73±3114.66 | 22.41±7.18 | 0.83±0.29 | 0.03±0.01 | 1187.49±527.46 | 36.26±9.55 | 6.64±1.13 | 10.91±1.29 |
|  | Free | 266.00±17.58 | 342.14±48.47 | 359.21±50.46 | 9.65±2.5 | 0.83±0.29 | 0.07±0.02 | 20854.88±9102.98 | 1457.74±245.74 | 2.63±0.47 | 4.36±0.52 |
| 360 mg/m^2^  (n=3) | Total | 9823.33±1432.7 | 17499.11±3841.42 | 17902.33±3930.32 | 14.28±2.52 | 0.67±0.29 | 0.05±0.01 | 732.56±85.77 | 36.70±9.70 | 5.72±1.57 | 7.33±1.97 |
|  | Free | 299.33±74.41 | 534.75±160.70 | 566.45±175.73 | 11.29±6.11 | 0.67±0.29 | 0.07±0.03 | 18832.60±9147.95 | 1185.25±351.49 | 6.38±5.55 | 9.49±8.51 |
| 390 mg/m^2^  (n=6) | Total | 12471.67±2652.91 | 21063.01±3288.48 | 21503.97±3236.20 | 15.57±5.43 | 0.82±0.27 | 0.05±0.02 | 641.24±323.57 | 27.33±6.31 | 5.75±1.50 | 7.48±2.41 |
|  | Free | 412.83±109.09 | 566.96±103.21 | 586.44±100.28 | 7.45±1.19 | 0.82±0.27 | 0.09±0.01 | 11088.25±4474.78 | 1007.58±249.97 | 2.69±0.34 | 3.83±0.75 |

**AUC**,area under the plasma concentration-time curve ;**C_max,_** maximum observed plasma concentration;**CL**,total body clearance; **t_1/2_**,terminal elimination half-life;**T_max，_**time of maximum observed plasma concentration;**λz**,terminal elimination rate constant;**Vd**,apparent volume of distribution; **MRT**_,_mean residence time.

**Supplementary Table 10. Linear pharmacokinetic analysis (C_max_, AUC_0-t_, AUC_0-∞_) for total paclitaxel and free paclitaxel**

|  | PK parameters | PK interval (%) | Regression equation | Slope | | Linear PK* |
| --- | --- | --- | --- | --- | --- | --- |
|  |  |  |  | Point estimate(%) | 90% CI (%) |  |
| Total paclitaxel | C_max_(ng/mL) | 72.15-127.85 | Y=0.93*x+3.38 | 92.63 | (56.05,129.21) | No |
|  | AUC_0-t_(ng·h/mL) |  | Y=1.15*x+2.47 | 115.35 | (79.27,151.42) | No |
|  | AUC_0-∞_(ng·h/mL) |  | Y=1.11*x+2.79 | 110.77 | (76.41,145.14) | No |
| Free paclitaxel | C_max_(ng/mL) |  | Y=1.06*x-0.94 | 106.33 | (67.13,145.52) | No |
|  | AUC_0-t_(ng·h/mL) |  | Y=1.39*x-2.67 | 139.33 | (103.92,174.74) | No |
|  | AUC_0-∞_(ng·h/mL) |  | Y=1.36*x-2.41 | 135.94 | (101.10,170.78) | No |

**AUC**,area under the plasma concentration-time curve ;**C_max_**, maximum observed plasma concentration;**PK**, pharmacokinetics**.**

*If the confidence interval completely falls within the judgment interval, it can be considered that the PK parameter is linear with respect to the administered dose.
