# Supplementary figures and images for: Preclinical Development and Phase I Study of ZSYY001, a Polymeric Micellar Paclitaxel for Advanced Solid Tumor
Source: Cancer Med. 2025 Jul 22;14(14):e71039. doi: 10.1002/cam4.71039 (PMC12281596; doi:10.1002/cam4.71039)

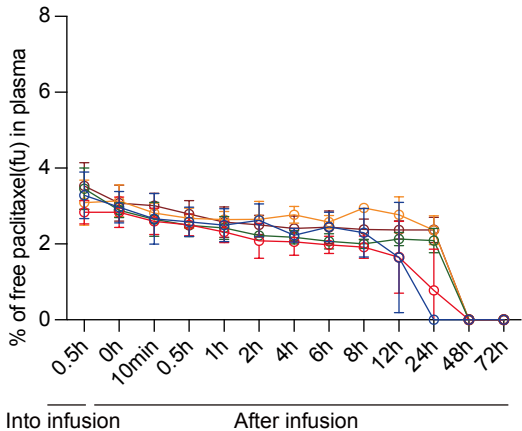

Supplement: Supplementary file 2 — Figure S1. Toxicity profile of ZSYY001 in healthy SD rats and beagle dogs. (A) Sixty rats were allocated to six groups [Ctrl: 0.9% normal saline; PM: 1000 mg/kg; Paclitaxel: 8 mg/kg; ZSYY001 (low): 50 mg/kg; ZSYY001 (medium): 100 mg/kg; ZSYY001 (high): 200 mg/kg]. Thirty‐six beagles were allocated to six groups [Ctrl: 0.9% normal saline; PM: 120 mg/kg; Paclitaxel: 8.0 mg/kg; ZSYY001 (low): 6 mg/kg; ZSYY001 (medium): 12 mg/kg; ZSYY001(high): 24 mg/kg]. All animals received the corresponding administration once followed by 4 weeks observation. Macroscopic characteristics after ZSYY001‐induced acute toxicity were observed. (B) Sixty SD rats were allocated to six groups [Ctrl: 0.9% normal saline; PM: 120 mg/kg; Paclitaxel:4 mg/kg; ZSYY001 (low): 10 mg/kg; ZSYY001 (medium): 20 mg/kg; ZSYY001(high): 30 mg/kg]. Thirty‐six beagles were allocated to six groups [Ctrl: 0.9% normal saline; PM: 60 mg/kg; Paclitaxel:2 mg/kg; ZSYY001 (low): 3 mg/kg; ZSYY001 (medium): 6 mg/kg; ZSYY001(high): 12 mg/kg]. All animals received the corresponding administration once a week for 4 consecutive weeks and then did not receive administration for 4 weeks. Macroscopic characteristics after ZSYY001‐induced sub‐acute toxicity were observed. Figure S2. Percentage of free paclitaxel(fu) in plasma at various times from start of the infusion. * Remove one isolated free drug measurement from the 360 mg/m2 dose group. [file CAM4-14-e71039-s001.zip › cam471039-sup-0003-Supinfo2@SFig-2.pdf]

A

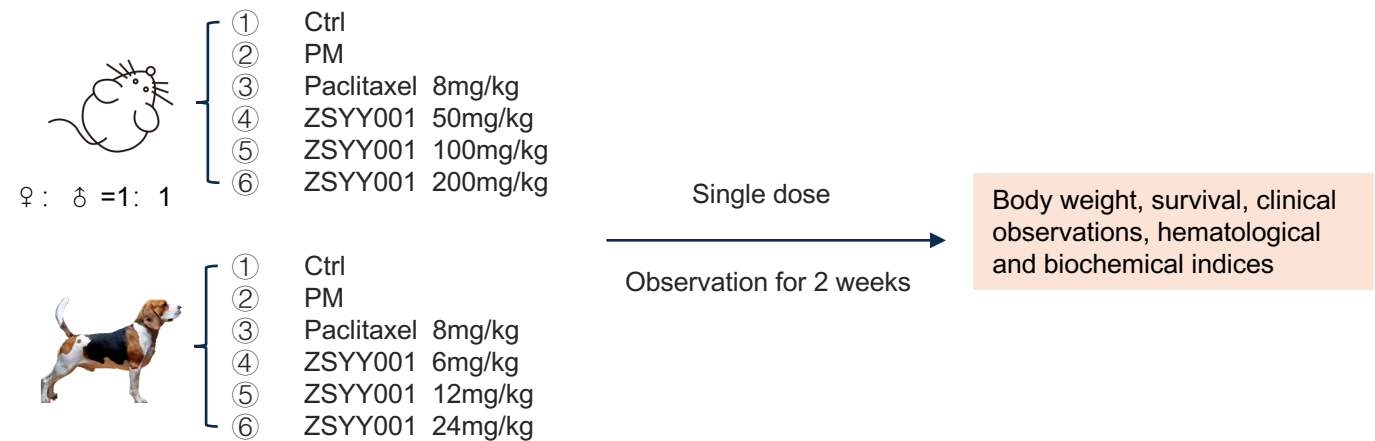

B

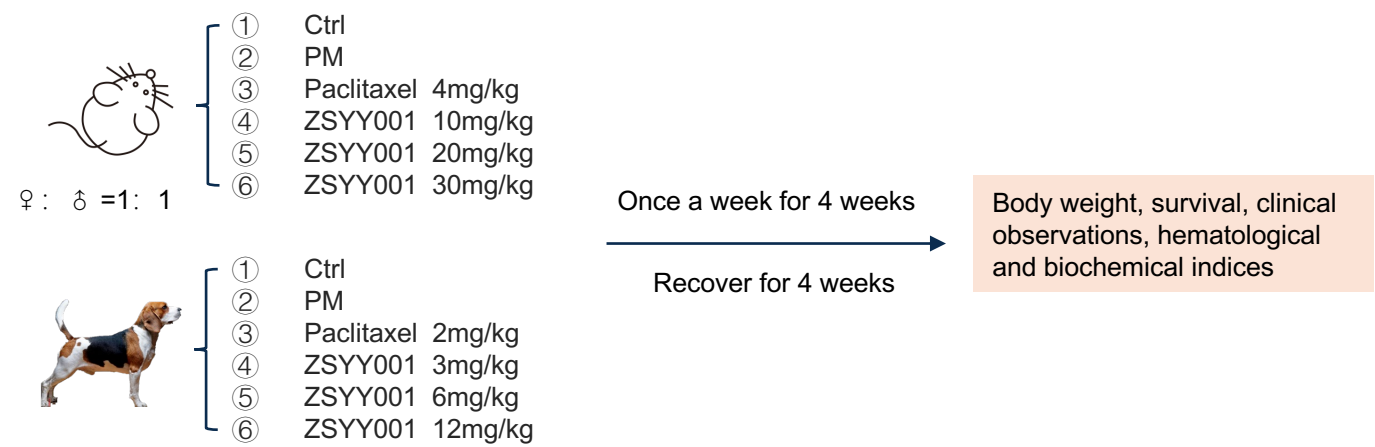

Supplement: Supplementary file 2 — Figure S1. Toxicity profile of ZSYY001 in healthy SD rats and beagle dogs. (A) Sixty rats were allocated to six groups [Ctrl: 0.9% normal saline; PM: 1000 mg/kg; Paclitaxel: 8 mg/kg; ZSYY001 (low): 50 mg/kg; ZSYY001 (medium): 100 mg/kg; ZSYY001 (high): 200 mg/kg]. Thirty‐six beagles were allocated to six groups [Ctrl: 0.9% normal saline; PM: 120 mg/kg; Paclitaxel: 8.0 mg/kg; ZSYY001 (low): 6 mg/kg; ZSYY001 (medium): 12 mg/kg; ZSYY001(high): 24 mg/kg]. All animals received the corresponding administration once followed by 4 weeks observation. Macroscopic characteristics after ZSYY001‐induced acute toxicity were observed. (B) Sixty SD rats were allocated to six groups [Ctrl: 0.9% normal saline; PM: 120 mg/kg; Paclitaxel:4 mg/kg; ZSYY001 (low): 10 mg/kg; ZSYY001 (medium): 20 mg/kg; ZSYY001(high): 30 mg/kg]. Thirty‐six beagles were allocated to six groups [Ctrl: 0.9% normal saline; PM: 60 mg/kg; Paclitaxel:2 mg/kg; ZSYY001 (low): 3 mg/kg; ZSYY001 (medium): 6 mg/kg; ZSYY001(high): 12 mg/kg]. All animals received the corresponding administration once a week for 4 consecutive weeks and then did not receive administration for 4 weeks. Macroscopic characteristics after ZSYY001‐induced sub‐acute toxicity were observed. Figure S2. Percentage of free paclitaxel(fu) in plasma at various times from start of the infusion. * Remove one isolated free drug measurement from the 360 mg/m2 dose group. [file CAM4-14-e71039-s001.zip › cam471039-sup-0002-Supinfo1@SFig-1.pdf]
